# Deubiquitinase USP1 enhances CCAAT/enhancer-binding protein beta (C/EBPβ) stability and accelerates adipogenesis and lipid accumulation

**DOI:** 10.1038/s41419-023-06317-7

**Published:** 2023-11-27

**Authors:** Myung Sup Kim, Jung-Hwan Baek, JinAh Lee, Aneesh Sivaraman, Kyeong Lee, Kyung-Hee Chun

**Affiliations:** 1 Department of Biochemistry & Molecular Biology, Seoul, Republic of Korea; 2 Graduate School of Medical Science, Brain Korea 21 Project, Seoul, Republic of Korea; 3 Institute of Genetic Science, Yonsei University College of Medicine, 50-1 Yonsei-ro, Seodaemun-gu, Seoul, 03722 Republic of Korea; 4 College of Pharmacy, Dongguk University-Seoul, Goyang, 10326 Republic of Korea

**Keywords:** Metabolic disorders, Ubiquitylation

## Abstract

Dysregulation of the ubiquitin-proteasome system has been implicated in the pathogenesis of several metabolic disorders, including obesity, diabetes, and non-alcoholic fatty liver disease; however, the mechanisms controlling pathogenic metabolic disorders remain unclear. Transcription factor CCAAT/enhancer binding protein beta (C/EBPβ) regulates adipogenic genes. The study showed that the expression level of C/EBPβ is post-translationally regulated by the deubiquitinase ubiquitin-specific protease 1 (USP1) and that USP1 expression is remarkably upregulated during adipocyte differentiation and in the adipose tissue of mice fed a high-fat diet (HFD). We found that USP1 directly interacts with C/EBPβ. Knock-down of USP1 decreased C/EBPβ protein stability and increased its ubiquitination. Overexpression of USP1 regulates its protein stability and ubiquitination, whereas catalytic mutant of USP1 had no effect on them. It suggests that USP1 directly deubiquitinases C/EBPβ and increases the protein expression, leading to adipogenesis and lipid accumulation. Notably, the USP1-specific inhibitor ML323—originally developed to sensitize cancer cells to DNA-damaging agents—decreased adipocyte differentiation and lipid accumulation in 3T3-L1 cells without cytotoxicity. Oral gavage of ML323 was administered to HFD-fed mice, which showed weight loss and improvement in insulin and glucose sensitivity. Both fat mass and adipocyte size in white adipose tissues were significantly reduced by ML323 treatment, which also reduced the expression of genes involved in adipogenesis and inflammatory responses. ML323 also reduced lipid accumulation, hepatic triglycerides, free fatty acids, and macrophage infiltration in the livers of HFD-fed mice. Taken together, we suggest that USP1 plays an important role in adipogenesis by regulating C/EBPβ ubiquitination, and USP1-specific inhibitor ML323 is a potential treatment option and further study by ML323 is needed for clinical application for metabolic disorders.

## Introduction

Adipogenesis, the process by which pre-adipocytes become fully differentiated adipocytes, is a key mechanism for increasing fat mass and lipid accumulation [1]. These are important metabolic pathways to maintain energy homeostasis by sensing and storing excess nutrients. To maintain homeostasis in adipose tissues, adipocytes undergo dynamic remodeling in response to changes in nutritional status [2]. Adipose tissues synthesize and store energy in the form of triglycerides (TGs) for long-term use and free fatty acids (FFAs) are released from TGs when energy is required [3]. They also generate numerous adipokines and signaling molecules that regulate metabolism throughout the body [4]. As the onset of various diseases caused by obesity has become a global problem, studies on the role of adipose tissues are being actively conducted [1–3, 5]. For example, improper adipogenesis results in a sustained inflammatory response and elevated serum glucose and lipid levels, leading to lipotoxicity in other major organs, including the kidneys, heart, and liver [6]. Therefore, appropriate expansion of adipose tissue through adipogenesis reduces the inflammatory response and fibrosis, while maintaining the healthy metabolic function of adipose tissues.

In this study, we demonstrated the role of deubiquitinase in adipogenesis and lipid accumulation. We were interested in the mechanisms that regulate protein homeostasis in adipocyte, because aberrant regulation and accumulation of proteins could provoke improper adipogenesis and/or lipid levels. Dysregulation of deubiquitinase is one of the causes of disruption of protein homeostasis [7]. For example, irregularities in the ubiquitin-proteasome system can disrupt the cellular response to insulin signaling by contributing to insulin resistance. This is done by reducing the activity of insulin receptor substrate (IRS) proteins, which transmit the insulin receptor’s signal within the cell. Mice deficient in IRS2 are prone to develop diabetes with a decrease in β-cell mass [8, 9]. The ubiquitin-mediated degradation of PPARγ is associated with nonalcoholic fatty liver disease (NAFLD). Clinical trials have shown that PPARγ expression is increased in the liver of NAFLD patients. CHIP E3 ligase directly ubiquitinates PPARγ, which leads to reduced adipocyte differentiation [10, 11]. Furthermore, dysregulation of the ubiquitin-proteasome system is not limited to ubiquitin ligases, but has also gained much attention in the field of deubiquitinases. For instance, the absence of USP19 in SVFs of iWAT fails to promote lipid accumulation and transcription of adipogenic genes, resulting in improved insulin sensitivity [12]. In addition, USP7 plays an important role in 3T3-L1 cell differentiation and mouse adipose tissue by deubiquitinating the acetyltransferase Tip60, which constitutes a transcriptional co-regulatory complex that induces adipocyte differentiation. The inhibition of USP7 activity results in reduced adipogenesis [13, 14].

CCAAT/enhancer-binding protein (C/EBP) β is a transcription factor that plays an essential role in adipogenesis, the process by which undifferentiated precursor cells differentiate into mature fat cells. C/EBPβ plays an indispensable role throughout the entire adipogenesis process. Preadipocytes are first committed to adipocyte lineage. This process is triggered by numerous factors including hormones and growth factors. C/EBPβ is expressed early in this process and trans-activates other important transcription factors including C/EBPα and PPARγ. Suppressing C/EBPβ leads to a reduction in adipogenesis, while overexpression of C/EBPβ prompts adipocyte differentiation even in the absence of other inducers [15]. C/EBPβ contributes to cell proliferation during the phase of mitotic clonal expansion, leading to an increase in preadipocytes numbers [16, 17]. Despite the critical role of C/EBPβ in adipogenesis, its cellular homeostasis remains enigmatic. Presently, it is established that ubiquitin ligase COP1 is responsible for ubiquitinating C/EBPβ, but this understanding primarily pertains to microglia [18].

In this study, we screened the expression levels of deubiquitinase before and after 3T3L1 adipocyte differentiation, and found the ubiquitin-specific protease 1 (USP1) expression was increased after adipocyte differentiation. USP1 is known to play a critical role in Fanconi anemia, complementation group A by deubiquitinating mono-ubiquitinated FANCD2 [19]. USP1 is involved in PCNA-mediated translesion synthesis via deubiquitination of monoubiquitinated PCNA [20]. PCNA is a novel factor that regulates translesion DNA synthesis [21, 22]. Interestingly, USP1 was reported to regulate glucose uptake and muscle atrophy via deubiquitination of AKT during fasting [23]. However, there are few studies relating metabolic diseases or obesity to the function of USP1. Therefore, we investigated the molecular mechanism of USP1 in adipogenesis and lipid accumulation.

## Results

### Expression level of USP1 is elevated in white adipose tissues of high-fat diet mice and 3T3-L1 cells

**Fig. 1:**
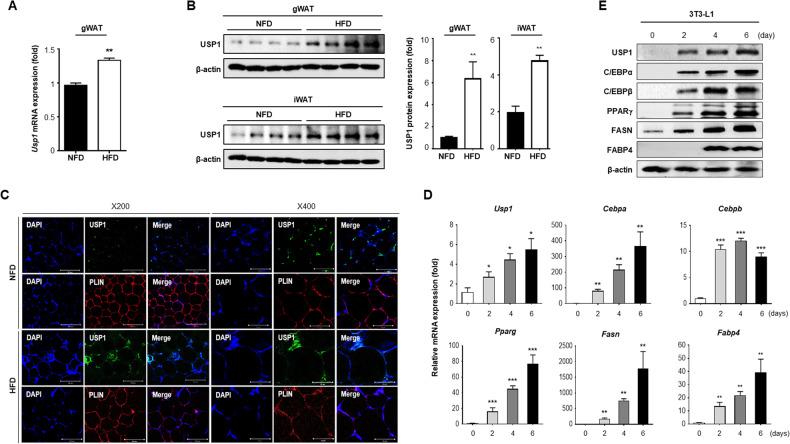
Protein and mRNA expression levels of USP1 in adipocyte differentiation. **A**, **B** mRNA and protein levels of USP1 in the adipose tissues from mice fed a normal-fat diet (NFD) or a high-fat diet (HFD) for 12 weeks. Statistical significance was determined by two-tailed unpaired *t* test. **C** Representative immunofluorescence images of gWAT from NFD or HFD mice for USP1. PLIN, the most abundant lipid droplet coat protein in adipocytes, was used as a marker for adipogenesis. **D** Quantitative RT-PCR analysis of mRNA for *Usp1* and adipocyte differentiation markers in 3T3-L1 cells. Results were normalized using β-actin. Statistical significance was determined by one-way ANOVA test. **E** Western blot analysis was used to assess protein levels of USP1 and adipocyte markers during adipocyte differentiation in 3T3-L1 cells. Results were normalized using β-actin. Data represent the mean ± SEM ^*^p < 0.05, ^**^p < 0.01, and ^***^p < 0.001 for day 0 vs. day 2, day 4, day 6.

In a preliminary study, RNA screening was performed to differentiate 3T3-L1 cells. The 3T3-L1 cells were harvested on days 0, 2, 4, and 6 after DMI induction. Changes in *Usp1* expression were also observed during adipocyte differentiation (data not shown). Therefore, the expression levels of *Usp1* in various tissues of normal mice were determined. Total RNA was extracted from major organs, including brown adipose tissue (BAT), gonadal white adipose tissue (gWAT), and inguinal white adipose tissue (iWAT), of 8-week-old C57BL/6 wild-type (WT) mice. According to qRT-PCR data, high levels of *Usp1* expression were detected in BAT and gWAT (Fig. S1). Next, the expression of *Usp1* was examined in white adipose tissues of obese mice. White adipose tissue of high-fat diet (HFD)-induced obese mice showed significantly higher levels of *Usp1* expression at both the mRNA and protein levels compared to control mice fed a normal-fat diet (NFD) (Fig. 1A–C). Obese mice showed higher USP1 expression levels in adipose tissues as well as livers than lean mice, suggesting that HFD increased USP1 expression throughout the body (Fig. S2). Expression levels of USP1 and levels of markers for adipocyte differentiation, such as C/EBPα, C/EBPβ, PPARγ, FASN, and FABP4, increased during the course of 3T3-L1 differentiation into adipocytes in vitro (Fig. 1D, E). Collectively, these results strongly support the hypothesis that USP1 plays an important role in adipogenesis and lipid accumulation.

### USP1-depleted 3T3-L1 cells exhibit reduced adipocyte differentiation

**Fig. 2:**
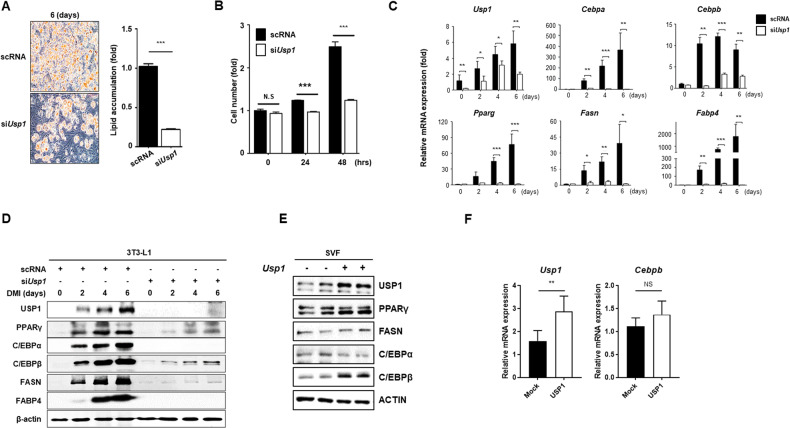
Knockdown of *Usp1* inhibits adipocyte differentiation. **A** Effect of *Usp1* knockdown on adipocyte differentiation. The 3T3-L1 cells were transfected with siRNA for 48 h before DMI induction and differentiated for 6 days. Lipid accumulation was measured using ORO staining. Stained dye was eluted with 100% isopropanol and measured at OD_500_. **B** A change of cell numbers during mitotic clonal expansion by *Usp1* knockdown in 3T3-L1 cells. Each sample was harvested 0, 24, and 48 h after DMI induction and cell numbers were counted using automatic cell counting machinery. **C**, **D** Quantitative RT-PCR and western blot analysis of adipogenic and lipogenic genes during adipocyte differentiation. **E**, **F** Protein and mRNA expression of USP1 and adipogenic factors by *Usp1* overexpression. Mouse adipose-derived stromal vascular fraction cells from gWAT were transfected with the *Usp1* plasmid. Results were normalized using β-actin. Data represent the mean ± SEM (*n* = 3 for each lane) ^*^p < 0.05, ^**^p < 0.01, and ^***^ *p* < 0.001 for scRNA vs. si*Usp1* and mock vs. *Usp1*. Statistical significance was determined by two-tailed unpaired *t* test.

To determine whether USP1 affects lipid accumulation in adipocytes, *Usp1* was knocked down in 3T3-L1 cells, and the cells were differentiated into adipocytes. ORO staining showed that lipid accumulation was significantly reduced by *Usp1* knock down of siRNA (Fig. 2A, Fig. S3). When 3T3-L1 cells differentiate into adipocytes, 3T3-L1 preadipocytes re-enter the cell cycle and undergo mitotic clonal expansion (MCE) [16, 24]. To identify the role of USP1 in MCE, *Usp1* expression was silenced in 3T3-L1 cells using siRNA, and the number of proliferating cells was measured. Notably, it was found that the proliferation of cells transfected with siRNA against *Usp1* was significantly reduced compared to that in the control group (Fig. 2B). Therefore, it was inferred that USP1 is important in the early stages of adipocyte differentiation. Next, whether the knockdown of *Usp1* affects the expression of genes regulating adipocyte differentiation and lipid metabolism was investigated. Expression levels of adipocyte markers were significantly reduced by *Usp1* knockdown during adipocyte differentiation in 3T3-L1 cells (Fig. 2C, D). Furthermore, the effect of USP1 on adipocyte differentiation was confirmed under USP1 overexpression conditions. The stromal vascular fraction (SVF) from the gWAT of 6-week-old mice was isolated and exogenously transfected with *Usp1*. Of the adipogenic transcription factors, *Usp1* overexpression resulted in a robust upregulation of C/EBPβ when compared to other transcription factors, such as PPARγ or C/EBPα, whereas *Usp1* overexpression did not lead to increased expression of *Cebpb* mRNA levels (Fig. 2E, F). These results suggested that USP1 is essential for adipocyte differentiation and USP1 is responsible for upregulating C/EBPβ protein levels.

### Inhibition of USP1 activity with the USP1-specific inhibitor attenuates adipocyte differentiation

**Fig. 3:**
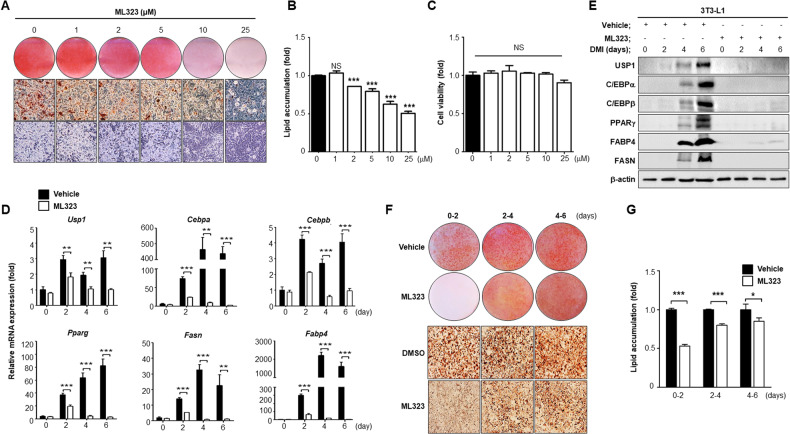
Adipocyte differentiation is attenuated in ML323-treated adipocytes. **A**, **B** The 3T3-L1 cells were treated (using a gradient of concentrations) with ML323 (0, 1, 2, 5, 10, and 25 µM) and stained with ORO 6 days after DMI induction. Cells were treated with ML323 every 48 h. **C** Confluent 3T3-L1 cells were treated with a range of ML323 concentration for 48 h. **D**, **E** Quantitative RT-PCR and western blot analysis of adipogenic and lipogenic genes during adipocyte differentiation. 3T3-L1 cells were treated with ML323 (25 µM) every 48 h along with DMI induction. Results were normalized using β-actin. **F**, **G** Differentiated 3T3-L1 cells stained with ORO dye on day 6. Cells were treated with ML323 (25 µM) on day 0–day 2, day 2–day 4, and day 4–day 6. Data represent the mean ± SEM (*n* = 3 for each lane) ^*^p < 0.05, ^**^p < 0.01, and ^***^p < 0.001 for vehicle vs. ML323 samples. Statistical significance was determined by one-way ANOVA test.

To investigate whether inhibition of USP1 activity also attenuates adipocyte differentiation, 3T3-L1 cells were treated with the USP1-specific inhibitor ML323 and differentiated for 6 days [25]. Adipocyte differentiation was significantly decreased with increasing ML323 concentration (Fig. 3A). Lipid accumulation was also reduced (Fig. 3B). A cell viability test was performed to confirm that ML323 was non-toxic to 3T3-L1 cells. There was no significant change in the cell number, regardless of the concentration of ML323 (Fig. 3C). Next, the inhibitory effects of ML323 on adipocyte differentiation were investigated. The mRNA and protein levels of PPARγ, C/EBPα, C/EBPβ, FASN, and FABP4 were significantly decreased by ML323 treatment, suggesting that USP1 inhibition by the USP1-specific inhibitor ML323 also reduced adipocyte differentiation (Fig. 3D, E). In addition, to understand the more accurate mechanism by which ML323 inhibits lipid accumulation, we treated 3T3-L1 cells with ML323 at different time points during the differentiation process. Notably, ML323 treatment at the early stage of differentiation significantly reduced lipid accumulation compared to that at a later stage (Fig. 3F, G). In summary, the inhibition of USP1 activity by ML323 treatment reduced adipocyte differentiation and lipid accumulation. Similar results were obtained when ML323 was used during the lipid accumulation phase.

### USP1 interacts with C/EBPβ and regulates its protein stability

**Fig. 4:**
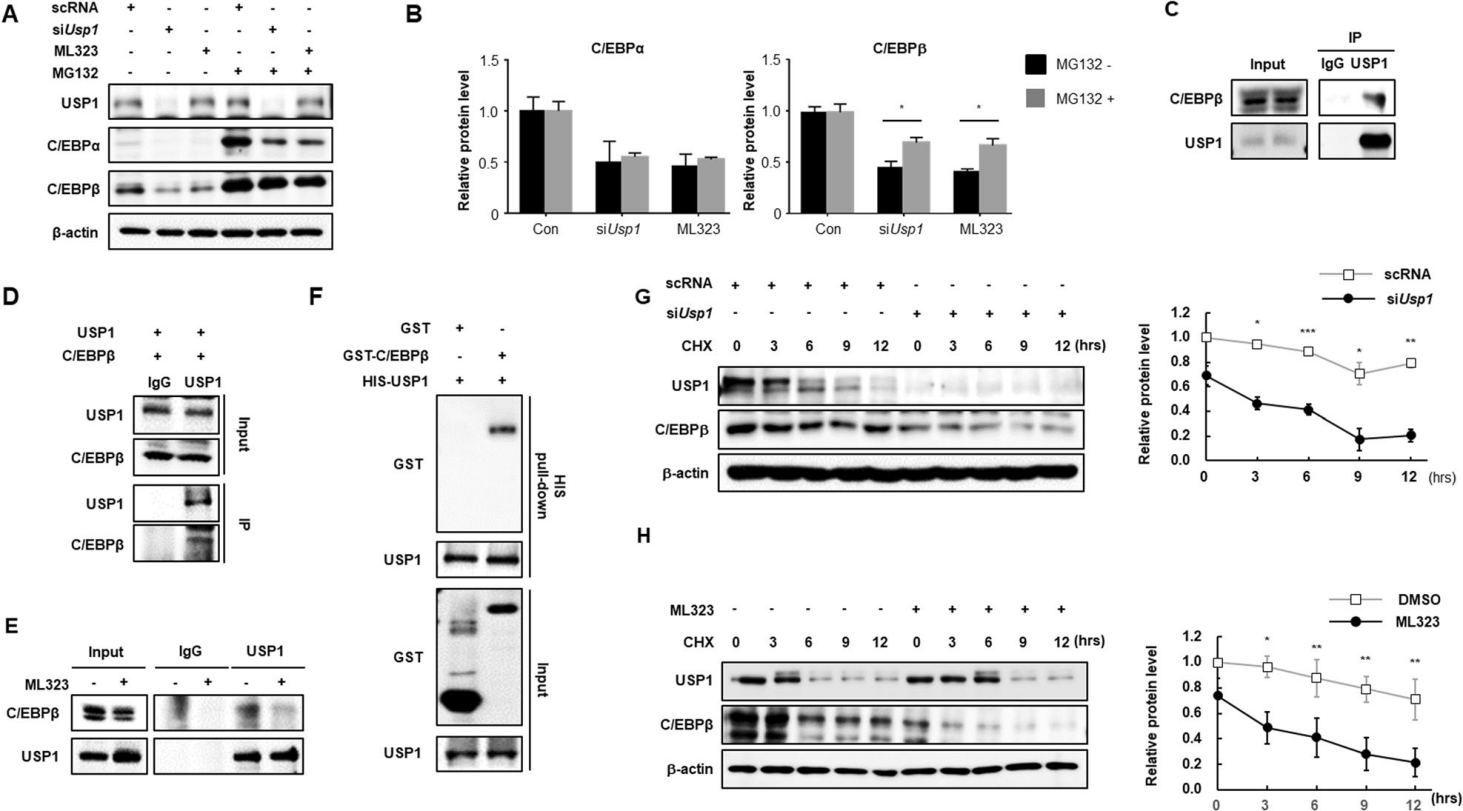
USP1 interacts with C/EBPβ and increases its protein stability. **A**, **B** Effects of *Usp1* knockdown on the protein stability of C/EBPα and C/EBPβ. AML12 cells were transfected with siRNA against *Usp1* or treated with ML323 for 48 h. Cells were then treated with or without 20 μM MG132 for 8 h. **C** Interaction between endogenous USP1 and C/EBPβ. The AML12 cells were immunoprecipitated using IgG and USP1 antibodies. **D** Immunoprecipitation of exogenous USP1 and C/EBPβ in HEK293 cells. Cell lysates were immunoprecipitated using IgG and USP1 antibodies and analyzed using western blotting. **E** Inhibition of interaction between endogenous USP1 and C/EBPβ by ML323. AML12 cells were treated with ML323 for 48 h and cell lysates were immunoprecipitated using IgG and USP1 antibodies. **F** HIS pull-down assay showing a direct interaction between USP1 and C/EBPβ. **G**, **H** Protein half-life of C/EBPβ by inhibition of USP1. AML12 cells were transfected with *Usp1* siRNA or treated with ML323 for 48 h. Results were normalized using β-actin. Data represent the mean ± SEM (*n* = 3 for each lane) ^*^P < 0.05, ^**^P < 0.01, and ^***^ *P* < 0.001 for scRNA vs. siUSP1. Statistical significance was determined by two-tailed unpaired *t* test.

Protein expression of transcription factors, such as PPARγ or C/EBPα, is not upregulated by *Usp1* overexpression (Fig. 2E), and as C/EBPβ is known to be regulated by the ubiquitin-proteasome pathway [26], whether USP1 can reverse proteasomal degradation of C/EBPβ was investigated. The proteasome inhibitor MG132 stabilized C/EBPβ, which is degraded in the absence of USP1 by siRNA or by inhibition of USP1 activity through ML323 treatment (Fig. 4A, Fig. S4). Both the C/EBP family members tested, C/EBPα and C/EBPβ, were stabilized by MG132; however, when protein levels were normalized to the baseline, C/EBPα did not show a reversal effect of USP1 inhibition on proteasomal degradation, as significantly as C/EBPβ did (Fig. 4B). Given that *Usp1* overexpression increased the expression of C/EBPβ, and not C/EBPα (Fig. 2E), we concluded that the protein stability of C/EBPβ is post-translationally regulated by the deubiquitinase USP1. Next the interactions between endogenous USP1 and C/EBPβ were examined (Fig. 4C). Immunoprecipitation assays confirmed their interaction when both proteins were overexpressed in HEK293 cells (Fig. 4D). Notably, ML323 treatment abrogated the interaction between USP1 and C/EBPβ (Fig. 4E). Inhibition of USP1 by ML323 appears to act as an allosteric modulator of the enzyme by binding to sites other than its catalytic site [27–31]. However, UAF1 forms a complex with USP1 to activate its deubiquitinating activity. ML323 is known to disrupt the interaction between USP1 and UAF1 [31], resulting in decreased interaction between USP1 and C/EBPβ. An in vitro His pull-down assay was performed to detect a direct interaction between USP1 and C/EBPβ (Fig. 4F). To investigate whether USP1 stabilizes C/EBPβ protein levels, AML12 cells were treated with cycloheximide. Cycloheximide enhanced C/EBPβ protein degradation when *Usp1* was knocked down by siRNA or inhibited by ML323 (Fig. 4G, H). *Cebpb* expression was not altered by siRNA knockdown of *Usp1* or chemical treatment (Fig. S5, S6). From these data, we concluded that USP1 can regulate the C/EBPβ protein levels via proteasomal degradation.

### USP1 regulates the protein stability of C/EBPβ by deubiquitination

**Fig. 5:**
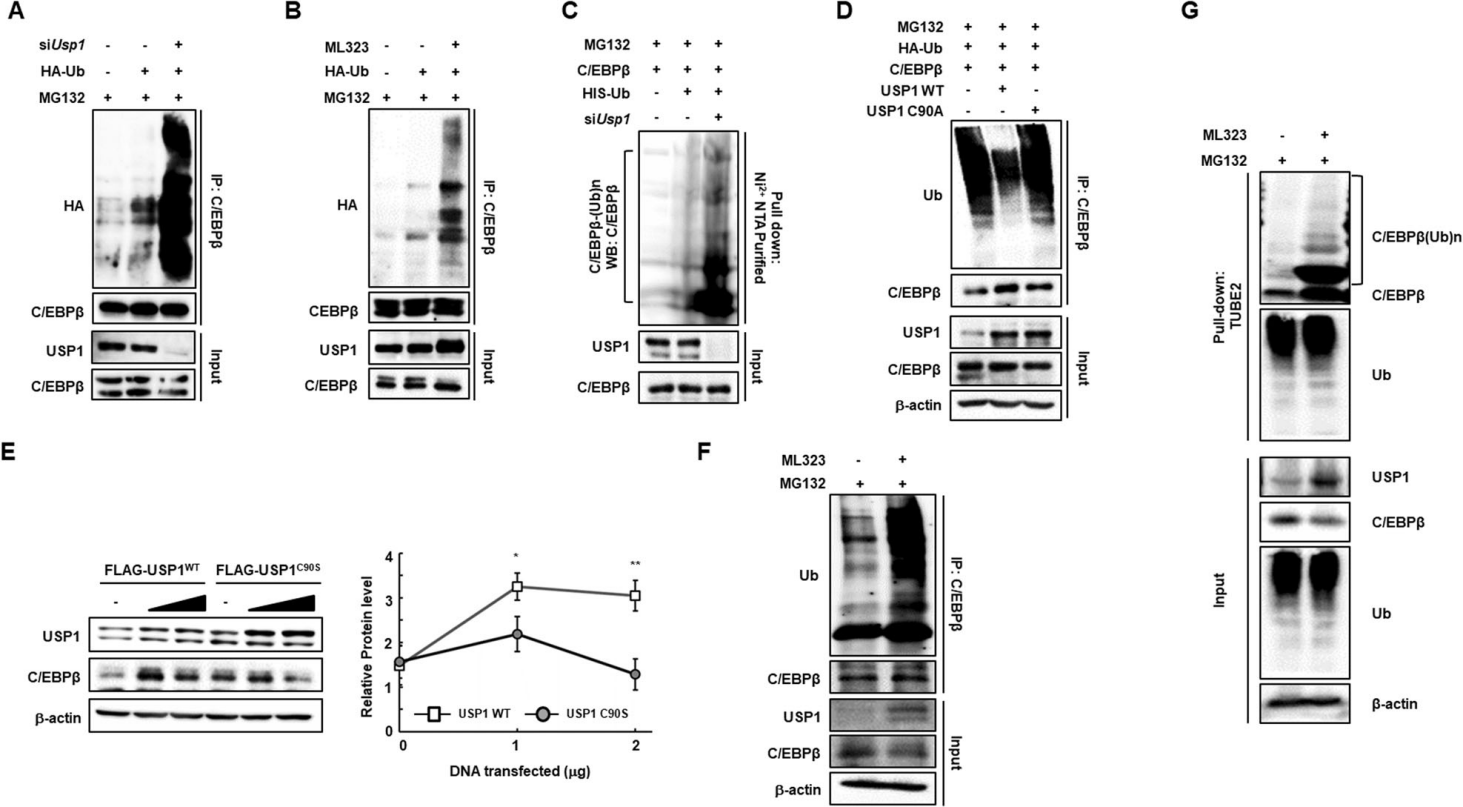
USP1 regulates the protein stability of C/EBPβ via deubiquitination. **A,** **B** Western blots of deubiquitination assays of C/EBPβ. AML12 cells were transfected with siRNA against *Usp1* or treated with ML323 followed by 20 μM MG132 for 8 h. **C** Western blots of Ni^2+^ NTA pull-down show the ubiquitination of overexpressed C/EBPβ with 20 μM MG132 for 8 h in AML12 cells **D** Western blots of deubiquitination assays in AML12 cells after transfection with either the USP1 WT and C90S mutant followed by 20 μM MG132 for 8 h. **E** Western blot analysis of USP1 WT and C90S mutant dose-dependent effects on C/EBPβ in AML12 cells. Cells were transiently transfected with USP1 WT and C90S mutant **F** Western blots of deubiquitination assays in primary adipocytes isolated from the gWAT. SVF cells obtained from the gWAT were differentiated into adipocytes and treated with or without ML323. **G** The total polyubiquitinated proteins in HEK293 cells were captured by TUBE2 resins. Cells were treated with ML323 for 48 h followed by 20 μM MG132 for 8 h. Data represent the mean ± SEM (*n* = 3 for each lane) ^*^P < 0.05 and ^**^P < 0.01 for USP1 WT vs. USP1 C90S. Statistical significance was determined by two-tailed unpaired *t* test.

After establishing the important role of USP1 in the regulation of C/EBPβ protein levels, its ubiquitin-mediated degradation and deubiquitination by USP1 were investigated. USP1 stabilizes the C/EBPβ protein via deubiquitination (Fig. 5A). Poly-ubiquitination marks leading to protein degradation of C/EBPβ were significantly enhanced by siRNA-mediated knockdown of *Usp1*. ML323-mediated inhibition of USP1 activity also resulted in the loss of C/EBPβ protein stability, while USP1 failed to regulate the protein stability of C/EBPα (Fig. 5B, Fig. S7). In addition, the His pull-down assay further confirmed that USP1 is the deubiquitinase for C/EBPβ (Fig. 5C). Overexpression of USP1 WT caused a decrease in C/EBPβ ubiquitination, leading to stabilized C/EBPβ protein levels, whereas USP1 C90S, a catalytically inactive form of USP1, did not (Fig. 5D, E). Furthermore, we investigated whether ML323 regulates C/EBPβ protein stability using SVF cells. We found that polyubiquitination of C/EBPβ caused by ML323 increased in isolated primary adipocytes, leading to the destabilization of C/EBPβ proteins (Fig. 5F). Consistent with these data, total polyubiquitinated proteins in HEK293 cells were captured using tandem ubiquitin-binding entities (TUBE 2), which are capable of detecting polyubiquitinated proteins. Inhibition of USP1 by ML323 resulted in an abundance of polyubiquitinated C/EBPβ proteins (Fig. 5G). Furthermore, we investigated the USP1-dependent regulation of protein stability of C/EBPβ in vivo, given that USP1 and C/EBPβ were upregulated in adipose tissues and livers of obese mice (Fig. 1A, B and Fig. S2). Mice were fed either a NFD or a HFD for 16 weeks followed by ML323 treatment. Adipose tissues and livers obtained from lean or obese mice were subjected to deubiquitination assays, which revealed that ML323 treatment led to the destabilization of C/EBPβ proteins (Fig. S8). Collectively, these results strongly indicated that C/EBPβ protein stability is regulated by USP1-mediated deubiquitination.

### Improved metabolic fitness of HFD-induced obese mice treated with USP1-specific inhibitors

**Fig. 6:**
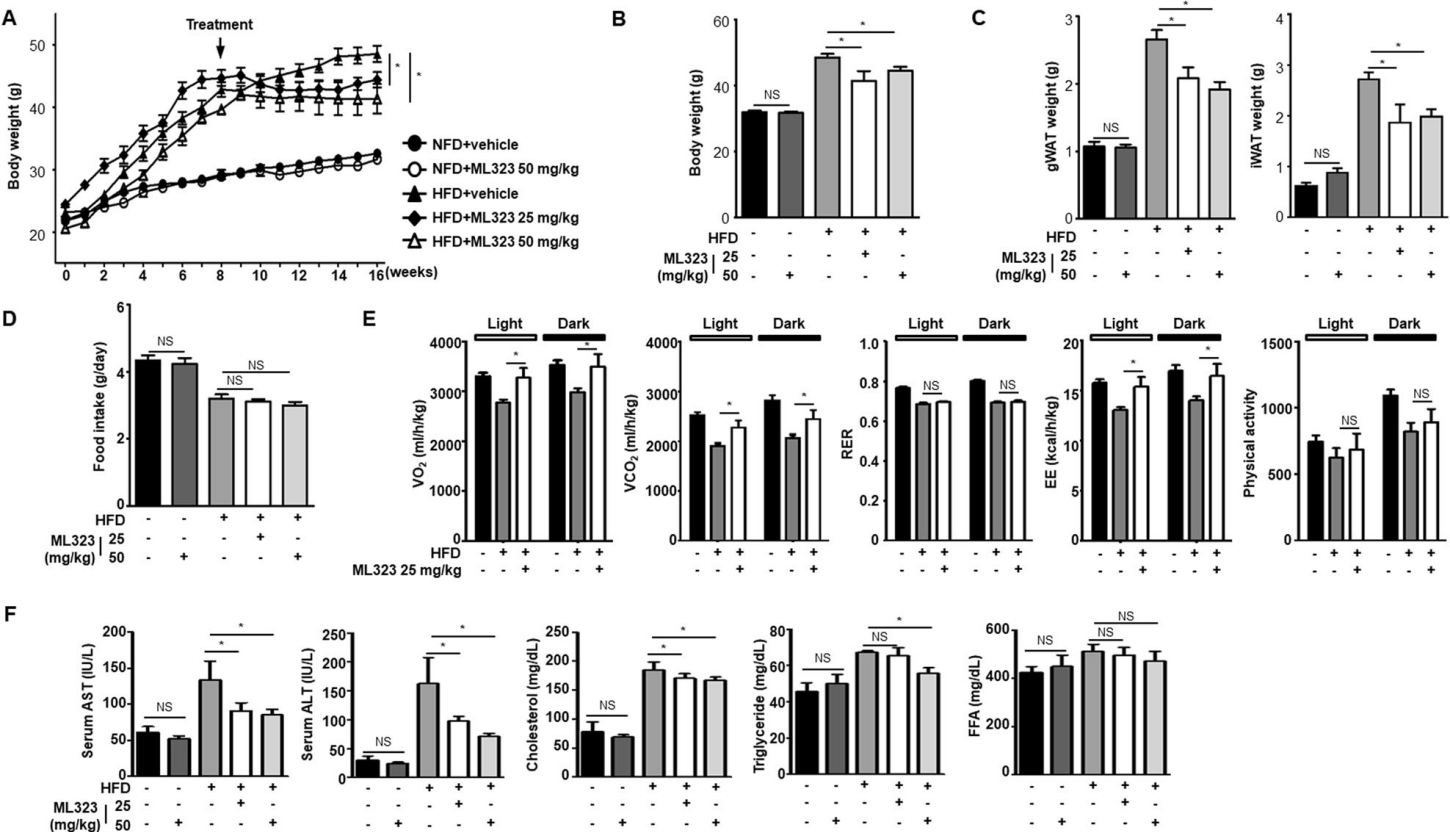
Improved metabolic fitness in ML323 treated HFD-induced obese mice. All mouse phenotypes were characterized 16 weeks after being fed a NFD or a HFD. **A**, **B** Body weight of mice treated with vehicle or ML323, fed an NFD or a HFD for 16 weeks with ML323 treatment starting on week 8. **C** Weight of gWAT and iWAT from vehicle-treated and ML323-treated mice. **D** Food intake of vehicle-treated and ML323-treated mice. **E** Metabolic parameters of vehicle-treated and ML323-treated mice. The metabolic parameters were measured for 1 week using a metabolic cage. **F** Serum concentrations of AST, ALT, cholesterol, triglyceride, and free fatty acid from vehicle-treated and ML323-treated mice. Data represent the mean ± SEM (*n* = 5 for each group) ^*^p < 0.05, ^**^p < 0.01, and ^***^p < 0.001 for vehicle-treated vs. ML323-treated mice. Statistical significance was determined by two-tailed unpaired *t* test.

To further investigate the in vivo role of USP1, 7-week-old WT mice (*n* = 5) were fed an HFD for 16 weeks to induce obesity. Eight weeks after HFD, oral gavage of the USP1-specific inhibitor ML323 was administered. The body weight analysis of mice fed HFD with ML323 showed that ML323-treated obese mice gained significantly less body weight than mice treated with the vehicle (Fig. 6A, B). There were no significant differences in body weight between NFD-fed lean mice (control group) with or without ML323 treatment. Despite similar food intake, obese mice treated with ML323 exhibited lower adipose tissue weights than vehicle-treated obese mice (Fig. 6C, D). In addition, mice were monitored in metabolic cages to measure metabolic parameters in vehicle-treated or ML323-treated mice (Fig. 6E). ML323-treated obese mice showed increased oxygen consumption and carbon dioxide production, indicating increased energy expenditure. However, ML323 treatment did not alter the physical activity of obese mice. ML323-treated obese mice consumed a similar number of calories from food intake, while consuming more calories than vehicle-treated control mice. Furthermore, biochemical variables such as aspartate aminotransferase (AST), alanine aminotransferase (ALT), cholesterol, triglyceride (TG), and free fatty acid (FFA) levels in the serum were measured. As expected, the HFD elevated all biochemical variables analyzed. ML323 treatment significantly reduced the obesity-induced increase in serum AST, ALT, cholesterol, and TG levels (Fig. 6F). ML323 treatment had minimal effects on serum FFA levels. Taken together, these results demonstrated that ML323 treatment of obese mice improves their metabolic fitness.

### Adipose tissues of ML323-treated mice exhibit enhanced fatty acid oxidation and reduced inflammation

**Fig. 7:**
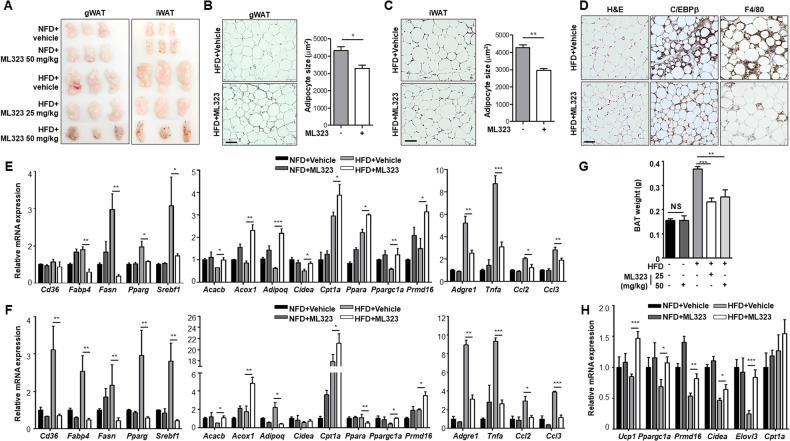
ML323-treated mice exhibit decreased adiposity and altered expression of lipid metabolism and inflammation. All mice phenotypes were characterized 16 weeks after NFD or HFD. **A** gWAT and iWAT from vehicle-treated and ML323-treated mice. **B**, **C** Adipocyte size of gWAT and iWAT sections stained with H&E. Size measurement was performed using ImageJ software. **D** Slide sections of the gWAT from vehicle-treated and ML323-treated mice fed an HFD. gWAT sections were stained with H&E and immunohistochemically stained using antibodies against C/EBPβ and F4/80. Scale bar = 100 μm. **E**, **F** Quantitative RT-PCR analysis of genes involved in fatty acid uptake, lipogenesis, fatty acid oxidation, and inflammation in gWAT and iWAT, respectively, from vehicle-treated and ML323-treated mice. **G** Weight of BAT from vehicle-treated and ML323-treated mice. **H** Quantitative RT-PCR analysis of genes involved in thermogenesis and fatty acid oxidation in BAT from vehicle-treated and ML323-treated mice. Data represent the mean ± SEM (*n* = 5 for each group) ^*^p < 0.05, ^**^p < 0.01, and ^***^p < 0.001 for vehicle-treated vs. ML323-treated mice. Statistical significance was determined by two-tailed unpaired *t* test.

The effect of ML323 on white adipose tissue sizes in ML323-treated obese mice were examined. The results showed that white adipose tissues of ML323-treated mice were smaller than those of vehicle-treated obese mice (Fig. 7A). H&E staining revealed that ML323-treated mice had smaller lipid droplet sizes and, consequently, smaller average adipocyte sizes (Fig. 7B, C). Histological analysis revealed that treatment with ML323 downregulated the expression of C/EBPβ and F4/80, which represent inflammatory macrophages in gWAT (Fig. 7D). The white adipose tissue gene expression levels associated with adipose tissue physiology and function were analyzed. Gene expression levels of the fatty acid transporters (*Cd36* and *Fabp4*) were first measured. ML323 treatment significantly reduced *Fabp4* expression in both gWAT (Fig. 7E) and iWAT (Fig. 7F), whereas *Cd36* had only a minor effect on gWAT. A fatty acid synthase (*Fasn*) and adipogenic transcription factors (*Pparg* and *Srebf1*) were significantly downregulated by ML323 treatment. Enzymes (*Acacb* and *Acox1*) promoting fatty acid oxidation were upregulated by ML323 treatment. Notably, *Adipoq* (adipokines involved in lipid metabolism and insulin sensitivity) and *Ppara* (transcription factor of lipid metabolism) expression was reduced by HFD in gWAT and was highly induced in ML323-treated obese mice, whereas HFD induced their expression in iWAT and was suppressed in ML323-treated obese mice, revealing the highly dynamic and complex nature of *Adipoq* and *Ppara* differential expression. Carnitine palmitoyltransferase 1A (*Cpt1*), a key enzyme for fatty acid oxidation in mitochondria, was highly induced by HFD and further enhanced by ML323 treatment. Previous studies have shown that browning of white adipose tissue inhibits diet-induced obesity in animals. Genes involved in adipose tissue browning (*Cidea*, *Ppargc1a*, and *Prdm16*) were significantly upregulated by ML323 treatment in obese mice. In addition, we observed that ML323 treatment significantly reduced the robust expression of inflammatory genes that were enhanced by HFD in both types of white adipose tissues (Fig. 7E, F). Brown adipose tissue enlarged by HFD also decreased in weight by ML323 treatment (Fig. 7G). The expression levels of genes that play a key role in adipose tissue browning decreased by HFD and increased by ML323 treatment (Fig. 7H). These data demonstrated that ML323 is a potent inhibitor of diet-induced obesity by promoting fatty acid beta-oxidation and white adipose tissue browning and preventing lipid metabolism and inflammation.

### ML323 improves hepatic steatosis and insulin sensitivity in obese mice

**Fig. 8:**
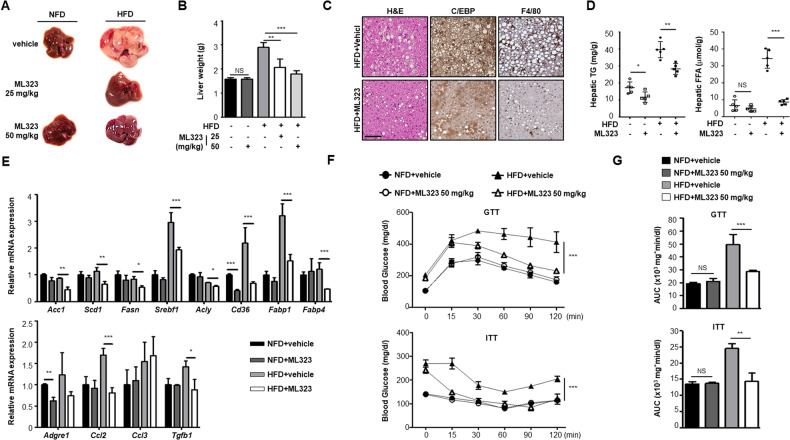
ML323-treated mice exhibit less hepatic steatosis and improve glucose homeostasis. All mice phenotypes were characterized 16 weeks after NFD or HFD. **A** Macroscopic view of liver from vehicle-treated and ML323-treated mice. **B** Liver weight of vehicle-treated and ML323-treated mice. **C** Slide sections of liver from vehicle-treated and ML323-treated mice fed an HFD diet. Liver sections were stained with H&E, and immunohistochemically stained using antibodies against C/EBPβ and F4/80. Scale bar = 100 μm. **D** Triglyceride and free fatty acid content in liver from vehicle-treated and ML323-treated mice. **E** Quantitative RT-PCR analysis of genes involved in fatty acid uptake, lipogenesis, and inflammation in livers from vehicle-treated and ML323-treated mice. **F**, **G** Glucose tolerance and insulin sensitivity tests. Blood glucose levels were measured at indicated time point. Data represent the mean ± SEM (*n* = 5 for each group) ^*^P < 0.05, ^**^P < 0.01, and ^***^ *P* < 0.001 for vehicle-treated vs. ML323-treated mice. Statistical significance was determined by two-tailed unpaired *t* test.

To investigate whether the inhibition of adipogenesis by ML323 affects the accumulation of ectopic lipids in non-adipose tissues, such as the liver, ML323 was administered to HFD-induced obese mice. Notably, obese mice treated with ML323 showed less hepatic steatosis (Fig. 8A) and lower liver weights than vehicle-treated obese mice (Fig. 8B). Histological analysis also revealed that ML323 alleviated hepatic steatosis and reduced C/EBPβ and F4/80 expression in the liver (Fig. 8C). ML323-treated obese mice also had lower TG and FFA content in the liver than vehicle-treated obese mice (Fig. 8D). We also found that the gene expression levels of enzymes involved in fatty acid synthesis (*Acc1*, *Scd1*, *Fasn*, and *Acly*) were reduced by ML323 treatment in obese mice. The adipogenic transcription factor (*Srebf1*) and fatty acid transporters (*Cd36*, *Fabp1* and *Fabp4*), which are strongly upregulated in fatty liver, were significantly downregulated by ML323 (Fig. 8E). Similarly, the expression of several upregulated inflammatory genes was suppressed by ML323. Moreover, the beneficial effects of ML323 on glucose homeostasis were evaluated to characterize metabolic phenotypes. ML323-treated HFD-fed mice showed improved glucose tolerance and insulin sensitivity compared to vehicle-treated obese mice. Blood glucose clearance and systemic insulin sensitivity were significantly improved in ML323-treated obese mice than those in vehicle-treated obese mice. (Fig. 8F, G). Collectively, these data demonstrated that HFD-induced severe lipid accumulation in the liver and can be reversed by ML323 treatment via the regulation of fatty acid synthesis and inflammation, resulting in improved systemic metabolism in the liver.

## Discussion

In this study, we demonstrated that USP1 has the potential to modulate metabolic diseases. To date, studies involving USP1 have been limited to the extent to which USP1 is important in promoting DNA damage repair in Fanconi anemia or regulating autophagy via ULK1 deubiqitination [32, 33]. Although studies have shown that USP1 regulates hepatic fibrosis and glucose metabolism [23, 34], this is the first study on USP1 in relation to adipogenesis. We confirmed that USP1 directly interacts with and deubiquitinates C/EBPβ, enhancing the stability of C/EBPβ in the regulation of adipogenesis. C/EBPβ is a family of transcriptional factors involved in cell proliferation and differentiation. CREB, activated by adipogenesis, rapidly induces the expression of C/EBPβ. C/EBPβ promotes the expression of C/EBPα and PPARγ, which are key adipogenic transcription factors involved in adipocyte differentiation [35].

During adipocyte differentiation, C/EBPβ is also highly regulated by various PTMs, including phosphorylation, acetylation, methylation, sumoylation, and ubiquitination [36]. C/EBPβ is phosphorylated by MAPKs, CDK2, and GSK3β, resulting in conformational changes [37, 38]. These conformational changes allow dimerization of C/EBPβ, enhancing its DNA-binding activity, and promoting adipogenesis. Phosphorylation of C/EBPβ is blocked by PRMT4/CARM1-mediated demethylation of C/EBPβ [39]. The protein inhibitor of activated STAT 1 (PIAS1) is a SUMO E3 ligase and sumoylates C/EBPβ. Sumoylation of C/EBPβ undergoes proteasomal degradation, owing to increased ubiquitination [40]. Conversely, SUMO-specific peptidase 2 (SENP2) promotes adipogenesis via desumoylation and stabilization of C/EBPβ [41]. In addition, COP1 is E3 ligase targeting C/EBPβ [18, 42]. COP1 suppresses neuroinflammation via proteasomal degradation of C/EBPβ. However, the E3 ligase that regulates C/EBPβ during adipogenesis has not been identified, and more, the deubiquitinase that targets C/EBPβ remains unknown.

Here, we determined that adipogenesis markers were markedly attenuated by USP1 knockdown during adipocyte differentiation at both the protein and mRNA expression levels. These results are sufficient to conclude that the transcriptional cascade of adipogenesis is not induced by a decrease in protein stability due to the inhibition of USP1 expression. In addition, reduced C/EBPβ expression is known to suppress adipogenesis and alleviates HFD-induced obesity in mice [43, 44]. Furthermore, administration of the USP1-specific inhibitor ML323 to mice fed an HFD effectively reduced fat mass, improved hepatic steatosis, and improved insulin sensitivity. In addition, ML323 did not alter serum AST and ALT levels in NFD-fed mice; however, serum AST and ALT concentrations in HFD-fed mice were significantly higher than those in NFD-fed mice, and ML323 significantly suppressed AST and ALT concentrations. Thus, ML323 did not induce harmful toxic effects in mice and was highly effective only in obese mice. As USP1 inhibition downregulates C/EBPβ protein levels, we asserted for the first time that USP1 inhibition is a potential therapeutic target against metabolic diseases, including obesity. USP1 binds to USP1-associated factor 1 (UAF1), which stimulates the deubiquitinase activity of USP1 [45]. ML323 exhibits reversible nanomolar inhibitory activity against the USP1/UAF1 complex with excellent selectivity [25, 27]. Pimozide and GW7647 were the first reported inhibitors of the USP1/UAF1 complex; these compounds are also known to regulate the activity of unrelated targets, whereas ML323 is known to specifically inhibit the activity of the USP1/UAF1 complex [46].

In conclusion, our results contribute to the current understanding of adipogenesis and confirm that USP1 is a first identified deubiquitinase of C/EBPβ. Moreover, we suggest the potential of the USP1 inhibitor ML323 in the treatment of obesity-related metabolic disorders and further study by ML323 is needed for clinical application.

## Materials and methods

### Cell culture and adipocyte differentiation

The 3T3-L1 cells were provided by Professor Jae-Woo Kim (Yonsei University). These cells were tested for mycoplasma contamination, maintained and differentiated as previously described [47]. The 3T3-L1 cells were maintained in Dulbecco’s Modified Eagle’s Medium (DMEM) (Welgene) supplemented with 10% bovine serum and antibiotics. Confluent 3T3-L1 cells were incubated for 48 h. Thereafter, the medium was replaced with DMEM supplemented with 10% fetal bovine serum (FBS), dexamethasone (1 μM), insulin (1 μg/mL) and isobutylmethylxanthine (520 μM). After 48 h, the medium was replaced with DMEM supplemented with 10% FBS and insulin (1 μg/mL). AML12 cells were cultured in DMEM: Ham’s F12 medium (1:1) with 0.005 mg/mL insulin, 0.005 mg/mL transferrin, 5 ng/mL selenium, 40 ng/mL dexamethasone, 10% FBS (Corning Cellgro), and 1% antibiotics (Invitrogen, Carlsbad, CA, USA) and maintained at 37 °C in a humidified incubator with a 5% CO_2_ atmosphere.

### Oil Red O staining

Differentiated 3T3-L1 cells were washed with DPBS and incubated in 10% formalin for 10 min. The cells were washed with distilled water. Thereafter, cells were washed with 60% isopropanol and completely dried. Oil Red O (ORO) stock solution (0.35 g/100 mL) was diluted in isopropanol to prepare a 60% ORO working solution. Dried cells were stained with ORO for 30 min and washed thrice with distilled water. Quantification of ORO staining was performed by ImageJ software.

### Transfection of small interfering RNA or DNA

Cells were transfected with mouse *Usp1* siRNA (20 nM) using Lipofectamine RNAimax (Invitrogen) according to manufacturer’s protocol. For 3T3-L1 cells, the medium was replaced with maintenance medium supplemented with 10% BS after 24 h. pcDNA3.1 vectors with *Cebpb* or *Usp1* was transfected using Lipofectamine 2000 (Invitrogen) or X-tremeGENE^TM^ HP DNA transfection reagent according to manufacturer’s protocol. Cells were harvested after 48 h.

### Western blot analysis

Cells were treated with 100 μM cycloheximide for up to 12 h or with 20 μM MG132 for 8 h. When necessary, 20 μM ML323 was treated for 24 to 48 h. Cell lysates were prepared using RIPA buffer (1% Triton X-100, 1% sodium deoxycholate, 0.1% sodium dodecyl sulfate, 150 mM NaCl, 50 mM Tris-HCl, pH 7.5, and 2 mM EDTA, pH 8.0). Cell lysates were incubated for 20 min on ice and centrifuged at 4 °C for 25 min at 13,200 rpm. The supernatant was subsequently transferred to a new microcentrifuge tube and its concentration was measured using a protein assay reagent (Thermo Scientific, Waltham, MA, USA). Protein samples were loaded in the wells of an SDS-PAGE gel and transferred to PVDF or nitrocellulose membranes (Merck Millipore, Billerica, MA, USA). Membranes were blocked with 5% skim milk or BSA for 1 hr. at room temperature. Thereafter, membranes were incubated with primary antibodies (USP1, PPARγ, C/EBPα, C/EBPβ, FASN, FABP4, β-actin from Santa Cruz Biotechnology, Dallas, TX, USA) overnight at 4 °C. Membranes were washed three times for 10 min with PBST and incubated with horseradish peroxidase-conjugated secondary antibodies (Bethyl Laboratories, Montgomery, TX, USA) for 1 h at room temperature. Then, membranes were washed three times for 10 min with PBST. FUSION SOLO (Vilber, Eberhardzell, Germany) was used for image detection, according to the manufacturer’s instructions. β-actin was used as the loading control.

### Immunoprecipitation

Cell lysates were extracted using an immunoprecipitation buffer. After centrifugation (4 °C for 25 min at 13,200 rpm), the supernatants were added to protein A/G agarose beads (Santa Cruz Biotechnology) and incubated at 4 °C for 30 min in a rotor for pre-clearing. After centrifugation (4 °C for 25 min at 13,200 rpm), anti-Flag beads (Sigma-Aldrich, St. Louis, Missouri, USA), anti-USP1, and normal IgG (negative control) were independently added to supernatants and were subsequently incubated overnight at 4 °C in a rotor. Immunoprecipitates were washed twice with immunoprecipitation buffer, added to 2× sodium dodecyl sulfate sample buffer, and boiled at 95 °C for 5 min. After centrifugation, supernatants were analyzed by western blotting. For deubiquitination assay using TUBE2-agarose pulldown assay, total ubiquitinated proteins were pulled down with 30 μl of TUBE2 beads for 2 h at 4 °C and followed by western blotting [48].

### HIS pull-down assay

For in vitro HIS pull-down assays, 2 μg of purified HIS-USP1 (R&D system, cat. E-564–050) was incubated with 5 μg of GST-C/EBPβ (ORIGENE, cat. TP305882) in binding buffer (pH 7.5 HEPES/KOH, 300 mM NaCl, 1 mM EDTA, 1% Triton X-100, 0.1% sodium deoxycholate, and 0.02% SDS) at 4 °C for 2 h and washed three times. The bound proteins were analyzed by western blotting with anti-USP1 and anti-GST antibodies [49].

### Ubiquitination assay

The ubiquitination assay was performed as mentioned previously [50]. Briefly, cells were lysed in phosphate-buffered saline (PBS), containing 5 mM N-ethylmaleimide. The cell lysate was diluted with non-denaturing lysis buffer, followed by fragmentation of the viscous chromatin complexes by passing the lysed suspension through a syringe and incubating on ice for 5 min. The dissociated cells were centrifuged at 13,200 rpm for 10 min at 4 °C followed by immunoprecipitation.

### RNA isolation and real-time PCR

Total RNA was prepared using an RNA-lysis reagent (Intron) according to the manufacturer’s instructions. cDNA (1 μg) was synthesized using a quantitative RT-PCR master mix (TOYOBO, Osaka, Japan). The following primers were used.

USP1, forward: 5′-GGACTGTGCAGCAGGAACAA-3′ and reverse: 5′-GGACTGTGCAGCAGGAACAA-3′, PPARγ, forward: 5′-AGGGCGATCTTGACAGGAAA-3′ and reverse: 5′-CGAAACTGGCACCCTTGAAA-3′, C/EBPα, forward: 5′-GACATCAGCGCCTACATCGA-3′ and reverse: 5′-TCGGCTGTGCTGGAAGAG-3′, C/EBPβ, forward: 5′-CCAGCTGAGCGACGAGTACA-3′ and reverse: 5′-GCTTGAACAAGTTCCGCAGG-3′, FASN, forward: 5′-TGGGTTCTAGCCAGCAGAGT-3′ and reverse: 5′-ACCACCAGAGACCGTTATGC-3′, FABP4, forward: 5′-CATCAGCGTAAATGGGGATT-3′ and reverse: 5′-TCGACTTTCCATCCCACTTC-3′, β-actin, forward: 5′-GGCTGTATTCCCCTCCATCG-3′ and reverse: 5′-CCAGTTGGTAACAATGCCATGT-3′. Real-time PCR was performed using SYBR Premix Ex Taq (Clontech Laboratories, Mountain View, CA, USA) with ABI instruments (Applied Biosystems Inc, Foster City, CA, USA). All results were normalized using β-actin.

### Measurement of cell number during mitotic clonal expansion

Cells were grown in 6-well culture plates and transfected with USP1 siRNAs. After 48 h (0 h sample), the cells were harvested, and the cell number was automatically counted (NANOENTEK). Similarly, DMI-treated 24- and 48-h samples were harvested at their time points, and cell numbers were automatically counted.

### Isolation of mouse preadipocyte

The SVF was isolated from the gonadal fat pads of a 6-week-old C57BL/6 mouse using a preadipocyte isolation kit (BioVision, Waltham, MA, USA). Briefly, fat pads were removed, minced, and digested using collagenase 37 °C for 1 h. The digested tissue lysates were filtered through a cell strainer and centrifuged for 10 min. The supernatants were removed from the pellets. The pellets were resuspended with RBC lysis buffer for 3 min, filtered through a cell strainer, and centrifuged for 10 min. The supernatants were removed from the pellets. The pellets were resuspended in pre-adipocyte medium and seeded on a cell culture plate.

### Cell viability assay

The 3T3-L1 cells were seeded in 12-well plates and incubated for 48 h until they reached confluence. Confluent 3T3-L1 cells were treated with ML323 for the indicated time periods. Cell viability was measured using EZ-Cytox (Daeil Lab Services, Korea) according to the manufacturer’s protocol.

### Mouse studies

Six-week-old C57BL/6 male mice were purchased from Orientbio. After 1 week of stabilization, mice were fed a HFD (Research Diets, Inc., New Brunswick, NJ, USA) containing 60% fat for 12–16 weeks (12-h light/12-h dark cycle). After 8 weeks on a HFD, mice were randomly selected for DMSO or ML323 groups and ML323 (25 and 50 mg/kg) was administered orally twice a week. Body weight was measured weekly. The animal studies were approved by the Yonsei University Health System Institutional Animal Care and Use Committee (2017-0022).

### Histological analysis and immunohistochemistry

gWAT, iWAT, and liver tissues were fixed in 10% formalin for 24 h and embedded in paraffin. Adipose tissue and liver paraffin sections were stained with H&E for morphological analysis.

Immunohistochemistry was performed on 5 µm formalin-fixed paraffin-embedded tissues. The tissue section was deparaffinized, rehydrated followed by heat-induced antigen retrieval using antigen retrieval buffer of pH 6.0 (Dako, Carpinteria, CA). After the quenching of endogenous peroxidase activity for 10 min, nonspecific binding was blocked with 1% BSA for 20 min at room temperature. The sections were incubated with anti-C/EBPβ (LSBio cat. LS-C344026) or anti F4/80 (Abcam cat. Ab6640) at a 1:500 dilution for overnight. The antigen-antibody reaction was detected with Dako EnVision^+^ Dual Link System-HRP (Dako) and DAB^+^ (3, 3′-diaminobenzidine; Dako) with counterstaining with hematoxylin. Negative controls, including immunoglobulin G (IgG) and omission of the primary antibody, were concurrently performed.

### Immunofluorescence

gWAT tissues were fixed in 10% formalin for 24 h and embedded in paraffin. Slides with 5 μm FFPE tissues was incubated with anti-PLIN (Abcam, cat. ab3526) or anti-USP1 (NOVUS, cat. NBP2-55036) antibodies at a 1:500 dilution overnight followed by incubation with FITC anti-rabbit, Cy7 anti-rabbit secondary antibodies (Invitrogen), and DAPI staining solution (Vector Laboratories, Burlingame, CA, USA). Images were analyzed using confocal microscope (LSM 700; Oberkochen, Germany).

### Glucose tolerance tests (GTT) and insulin sensitivity test (ITT)

For the glucose tolerance test, mice were fasted for 15 h and intraperitoneally injected with 1 g/kg glucose (Sigma-Aldrich). For the insulin tolerance test, mice were fasted for 6 h and intraperitoneally injected with 1 IU/kg (Sigma-Aldrich). Mice were restrained and tail was cleaned with 70% ethanol/gauze. A small amount of blood from the tail vein was extracted. The blood glucose levels of mice were measured 15, 30, 60, 90, and 120 min after injection [48].

### Statistical analysis

All quantified data were statistically analyzed by GraphPad Prism. For bar graphs, statistical significance was determined by two-tailed unpaired Student’s *t* test for two groups, or by one-way ANOVA test for multiple groups. Statistical differences *p* < 0.05 was considered statistically significant.

### Supplementary information

Supplementary Material

Original Data File

Reproducibility checklist

## Data Availability

Data supporting the present study are available from the corresponding author upon reasonable request.
