# Supplementary material for: Deubiquitinase USP1 enhances CCAAT/enhancer-binding protein beta (C/EBPβ) stability and accelerates adipogenesis and lipid accumulation
Source: Cell Death Dis. 2023 Nov 27;14(11):776. doi: 10.1038/s41419-023-06317-7 (PMC10681981; doi:10.1038/s41419-023-06317-7)
Supplement: Supplementary file 1 — Supplementary Material [file 41419_2023_6317_MOESM1_ESM.docx]

**Deubiquitinase USP1 enhances CCAAT/enhancer-binding protein beta (C/EBPβ) stability and accelerates adipogenesis and lipid accumulation**

**Running title**: USP1 deubiquitinases C/EBPβ and increases adipogenesis

Myung Sup Kim^1, 2, 3, *^, Jung-Hwan Baek^1, 2, 3, *^, JinAh Lee^1^, Aneesh Sivaraman^4^, Kyeong Lee^4^, Kyung-Hee Chun^1, 2, 3, #^

^1^Department of Biochemistry & Molecular Biology, ^2^Graduate School of Medical Science, Brain Korea 21 Project, ^3^Institute of Genetic Science, Yonsei University College of Medicine, 50-1 Yonsei-ro, Seodaemun-gu, Seoul, 03722, Republic of Korea, ^4^College of Pharmacy, Dongguk University-Seoul, Goyang 10326, Republic of Korea

*****These two authors contributed equally to this work.

**# Correspondence:** Kyung-Hee Chun Ph.D., Department of Biochemistry & Molecular Biology, Yonsei University College of Medicine, 50-1 Yonsei-ro, Seodaemun-gu, Seoul 03722, Republic of Korea. TEL: 82-2-2228-1699, FAX: 82-2-312-5041, E-mail: [khchun@yuhs.ac](mailto:khchun@yuhs.ac)

**Brain**

**Liver**

**Heart**

**Lung**

**BAT**

**iWAT**

**Kidney**

**Spleen**

**Muscle**

**gWAT**


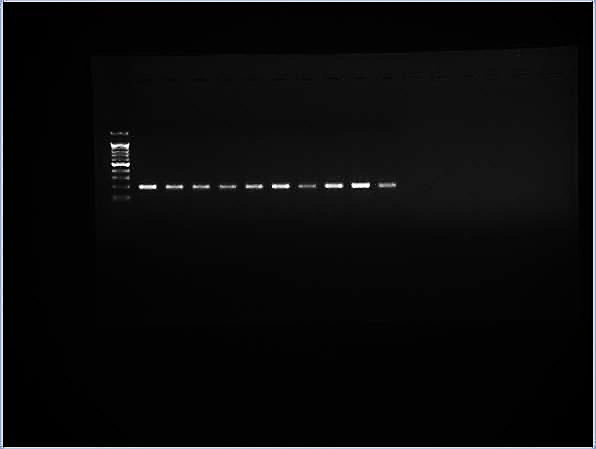

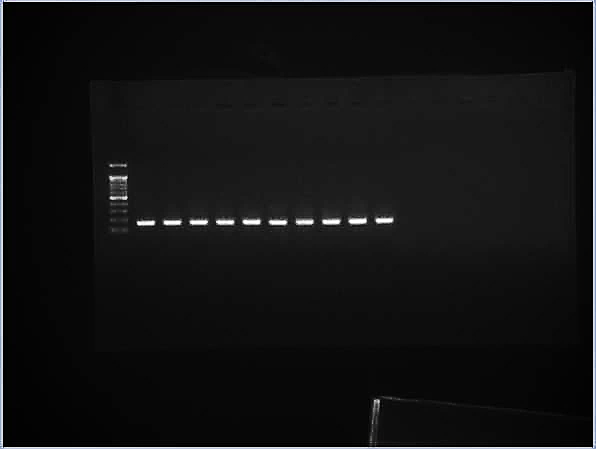


**USP1**

**β-actin**

**RT-PCR**

**Supplementary Fig. 1. The expression levels of *Usp1* from tissues of normal mice.** Representative image of *Usp1* mRNA levels in various tissues of three normal 8-week-old C57BL/6 mice. Results were normalized using β-actin. Each experiment was repeated more than three times independently.


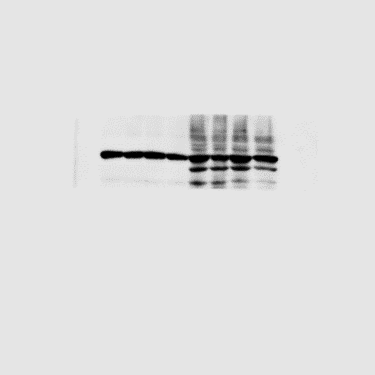

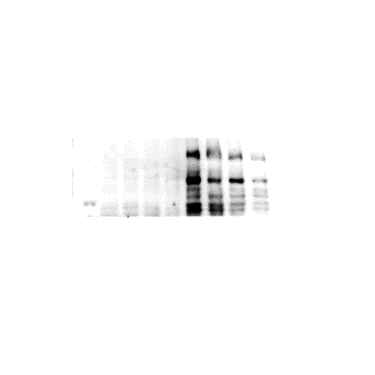


**Liver**

**NFD**

**HFD**

**USP1**

**β-actin**

**Supplementary Fig. 2. Elevated USP1 expression levels in livers from HFD-fed mice.** Western blot images of USP1 protein levels from liver tissues after 12 weeks of NFD or HFD. Results were normalized using β-actin.


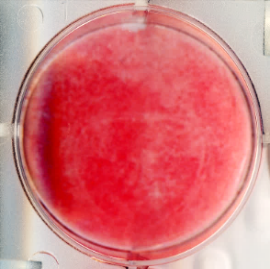

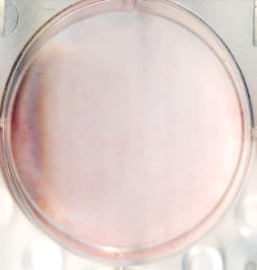

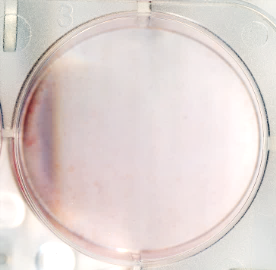

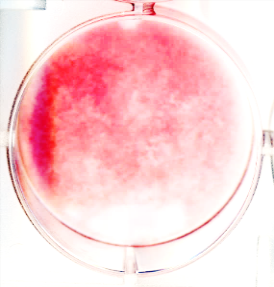


**NC**

**siUSP1#1**

**siUSP1#2**

**siUSP1#3**

**Lipid accumulation (fold)**

**-**

**#1**

**#2**

**#3**

**siRNA;**

**0**

**1.0**

**1.2**

**0.4**


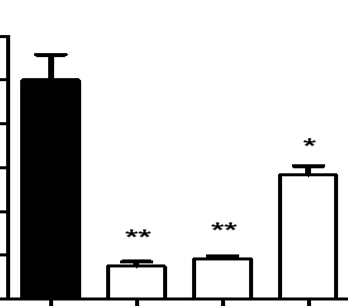


**0.2**

**0.8**

**0.6**

**Supplementary Fig. 3. Reduced lipid accumulation by siRNA mediated *Usp1* knock down was confirmed by ORO staining.** 3T3-L1 cells were transfected with siRNA for 48 hrs followed by DMI induction and differentiation for 6 days. Each experiment was repeated independently at least three times. Statistical significance was determined by two-tailed unpaired t test.


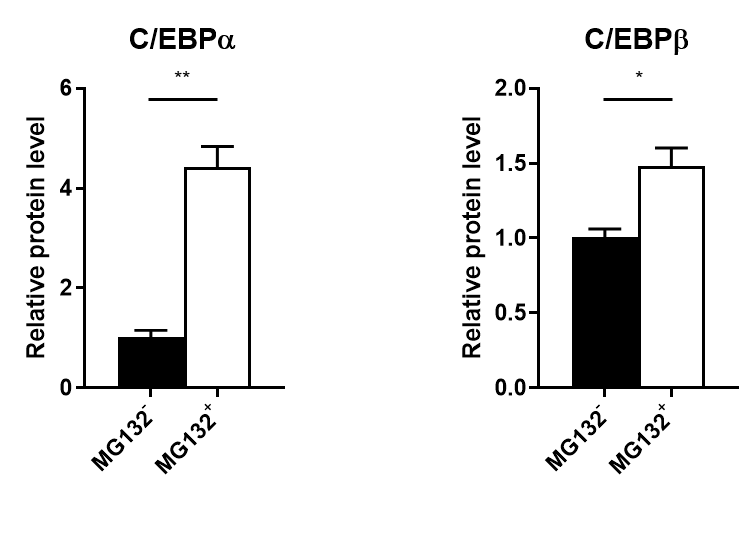


**Supplementary Fig. 4. Quantification of relative protein expression in response to MG132.** AML12 cells were treated with 20 μM MG132 for 8 hrs and normalized to DMSO treated control samples. Each experiment was independently repeated more than three times. Statistical significance was determined by two-tailed unpaired t test.


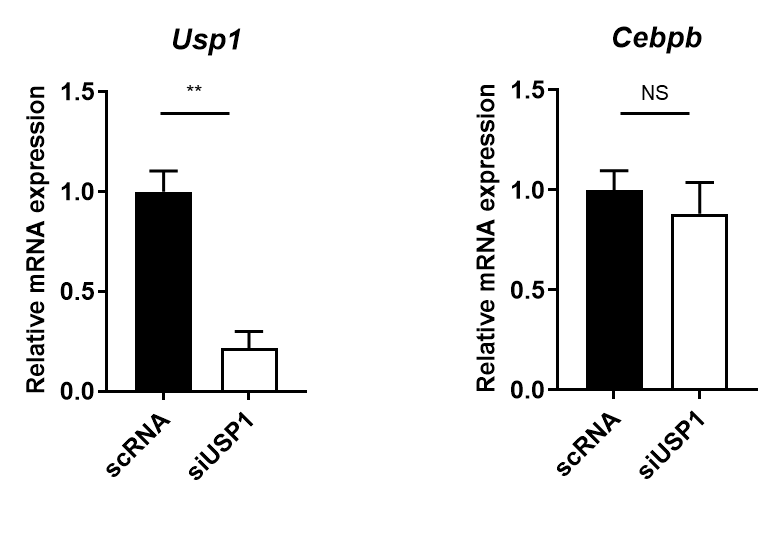


**Supplementary Fig. 5. Relative expression levels of *Usp1* and *Cebpb* by siRNA knockdown of *Usp1*.** Expression levels of *Usp1* and *Cebpb* from total mRNA isolated from AML12 cells. AML12 cells were transfected with siRNA against *Usp1* for at least 48 hrs. Statistical significance was determined by two-tailed unpaired t test.


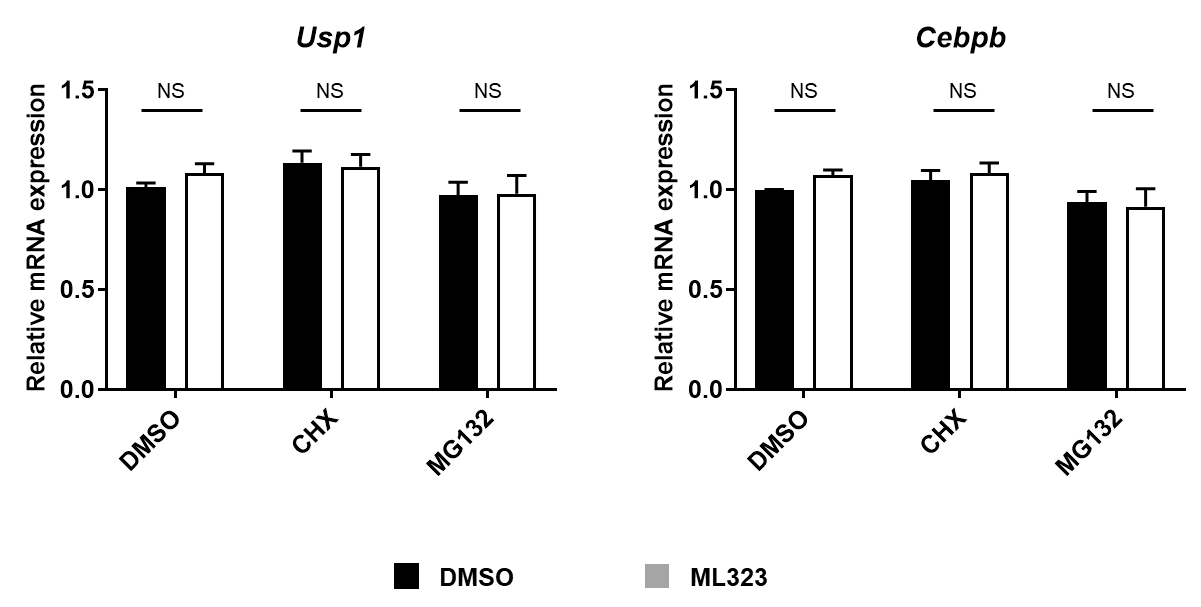


**Supplementary Fig. 6. Relative expression levels of *Usp1* and *Cebpb* when treated with cycloheximide, MG132, or ML323.** Expression levels of *Usp1* and *Cebpb* from total mRNA isolated from AML12 cells. AML12 cells were treated with 100 μM cycloheximide or 20 μM MG132 for 8 hrs in the presence of 20 μM ML323, and normalized to β-actin. Statistical significance was determined by two-tailed unpaired t test.

**
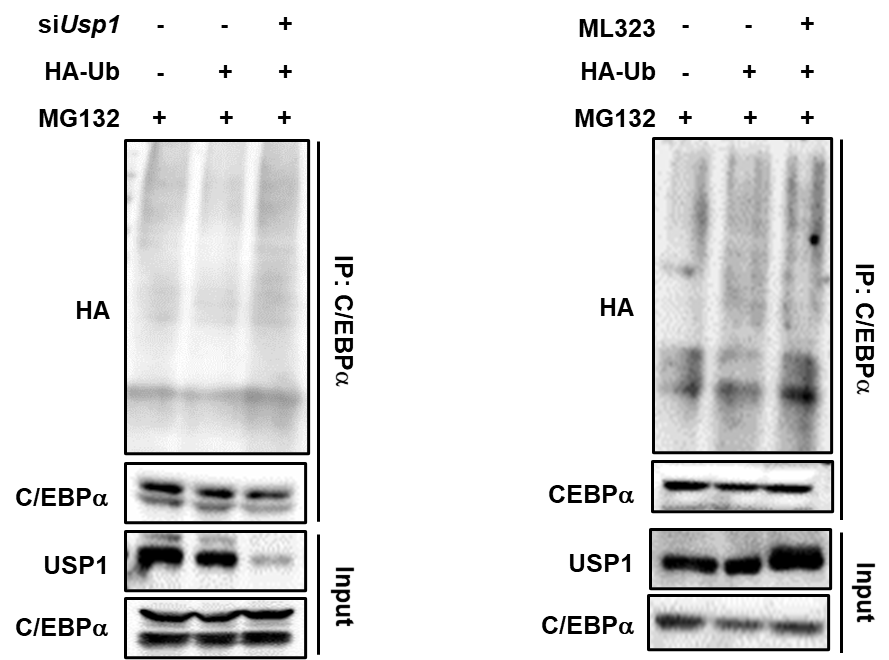
**

**Supplementary Fig. 7. Deubiquitination assays of C/EBPα in response to *Usp1* siRNA or ML323 treatment.** AML12 cells were transfected with siRNA against *Usp1* or treated with 20 μM ML323 for at least 48 hrs followed by 20 μM MG132 treatment for 8 hrs.

**
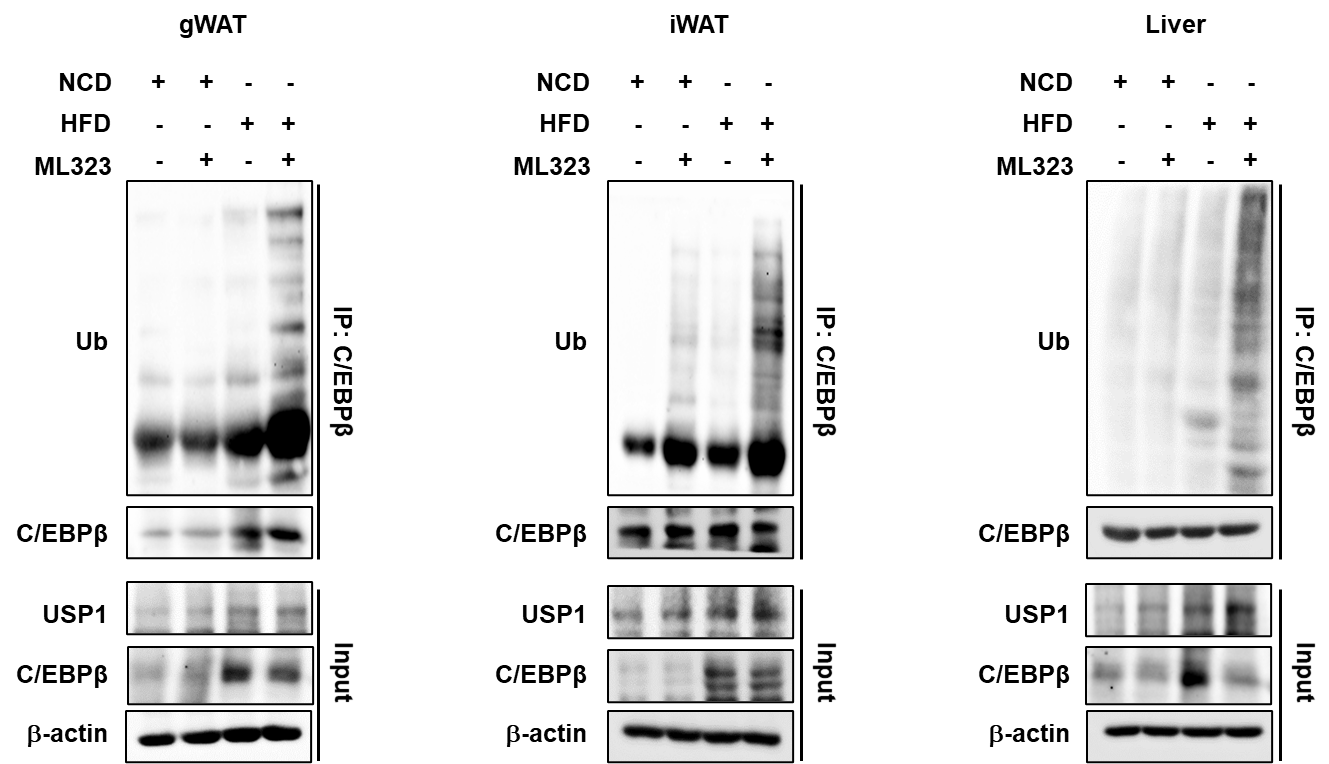
**

**Supplementary Fig. 8. Deubiquitination assays of C/EBPβ in ML323 treatment adipose tissues and livers obtained from a NFD or a HFD-fed mice.** After 16 weeks of a NFD or a HFD, 50 mg/kg ML323 was injected intraperitoneally more than 4 times (twice a weak). Adipose tissues and livers were harvested and subjected to deubiquitination assays to examine USP1-mediated stabilization of C/EBPβ proteins.
