# Supplementary material for: Deubiquitinase USP1 enhances CCAAT/enhancer-binding protein beta (C/EBPβ) stability and accelerates adipogenesis and lipid accumulation
Source: Cell Death Dis. 2023 Nov 27;14(11):776. doi: 10.1038/s41419-023-06317-7 (PMC10681981; doi:10.1038/s41419-023-06317-7)

Figure 1B

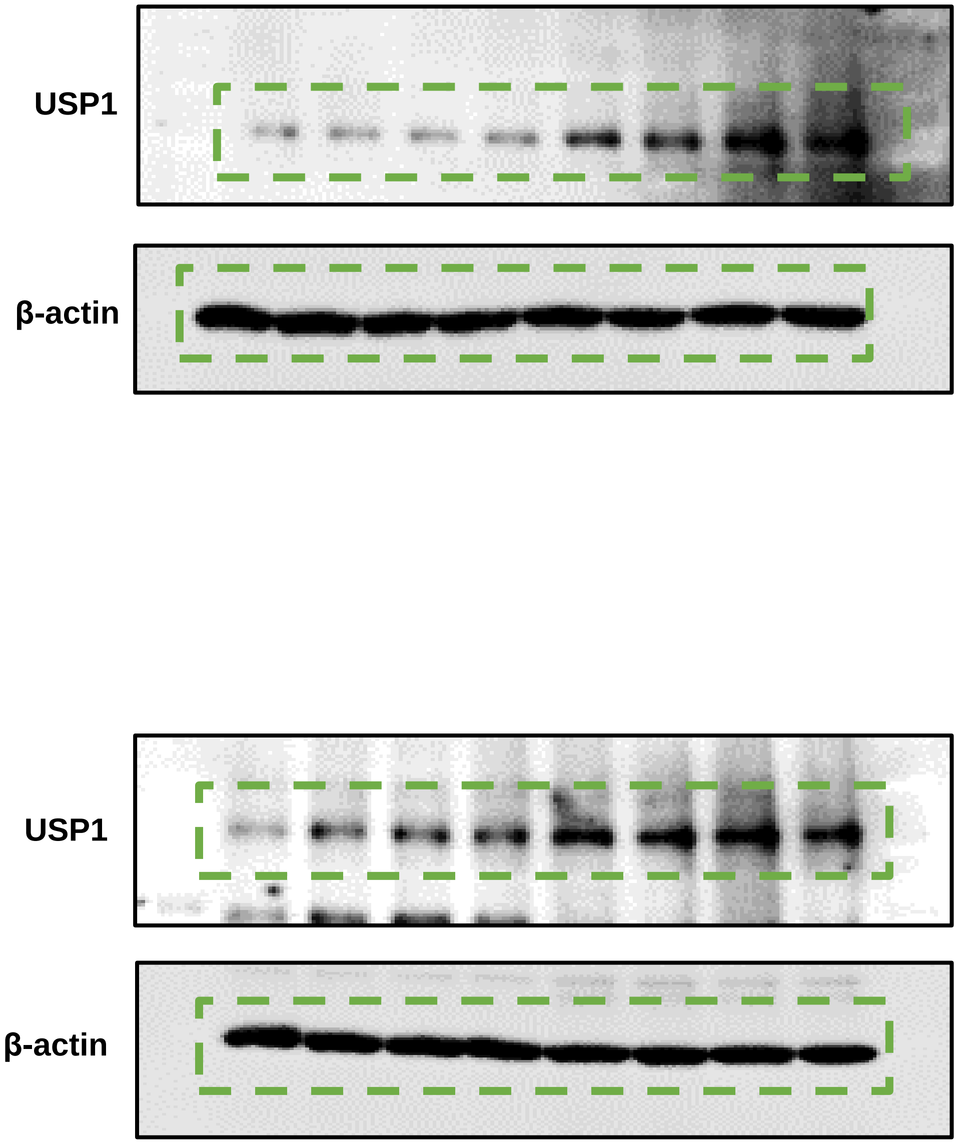

Figure 1E

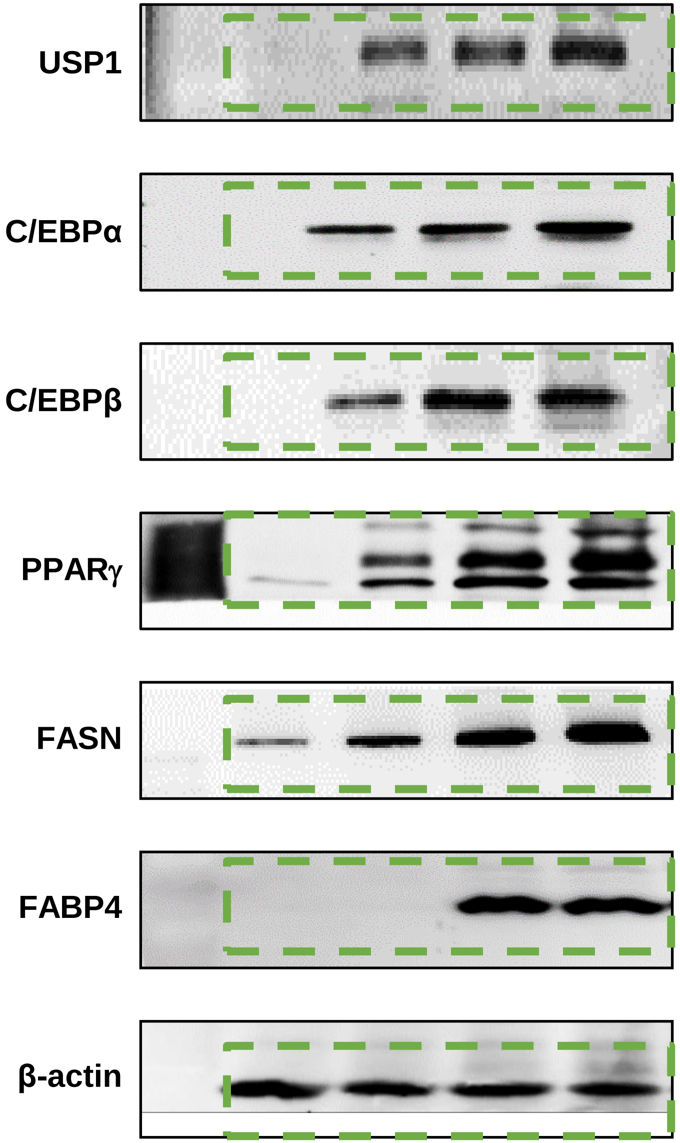

Figure 2D

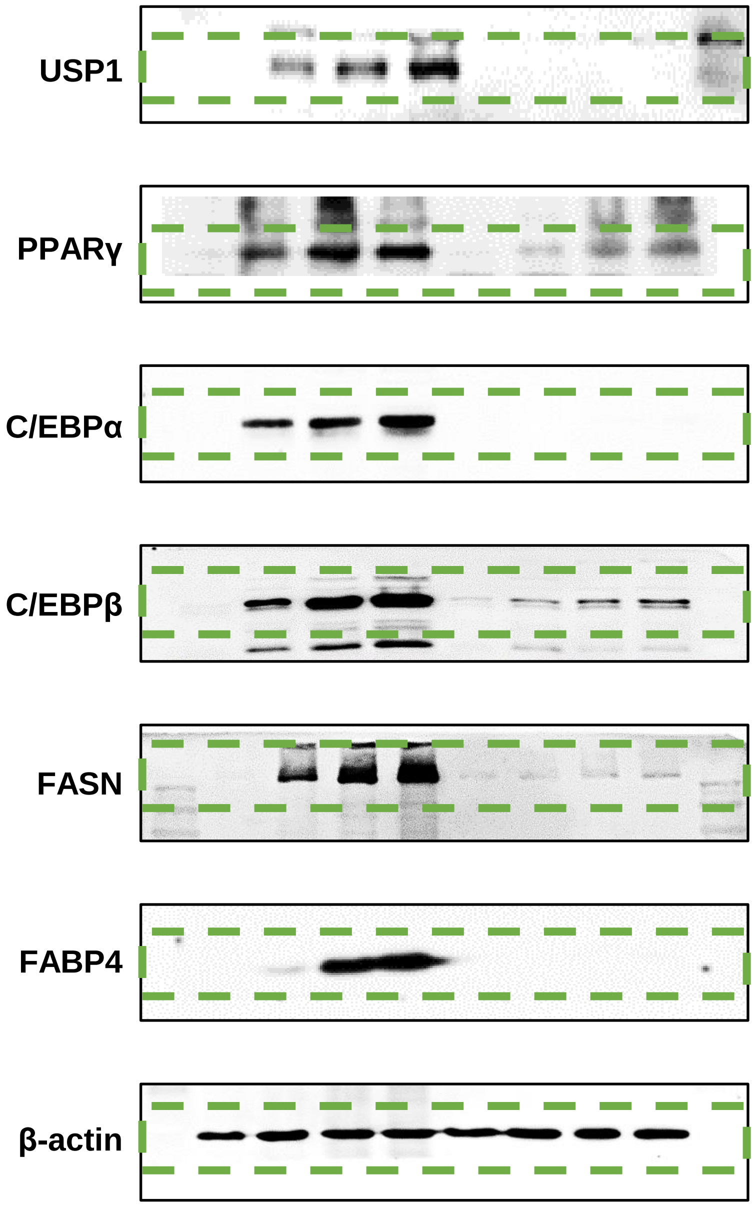

Figure 2E

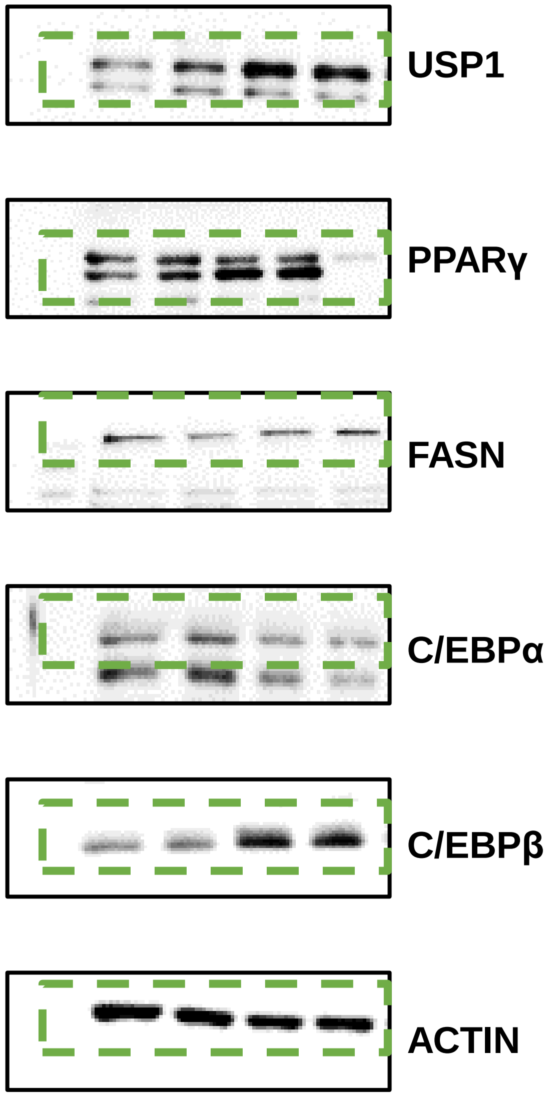

Figure 3E

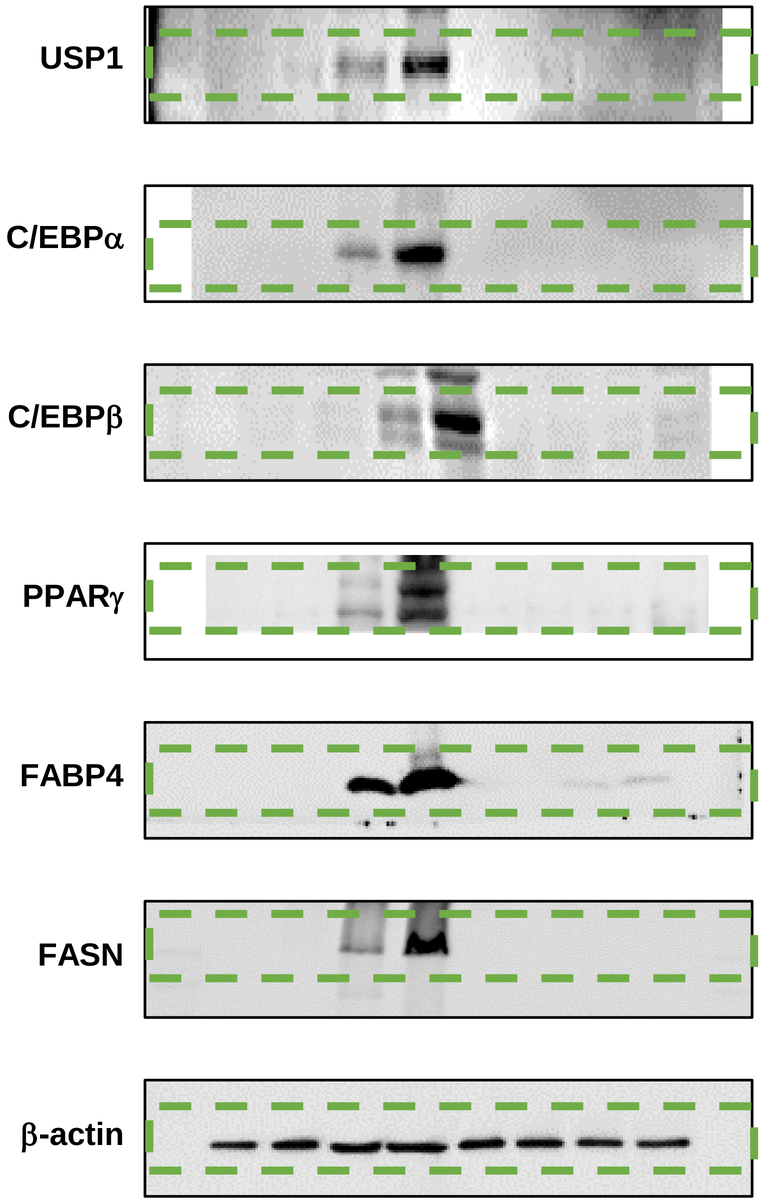

Figure 4A

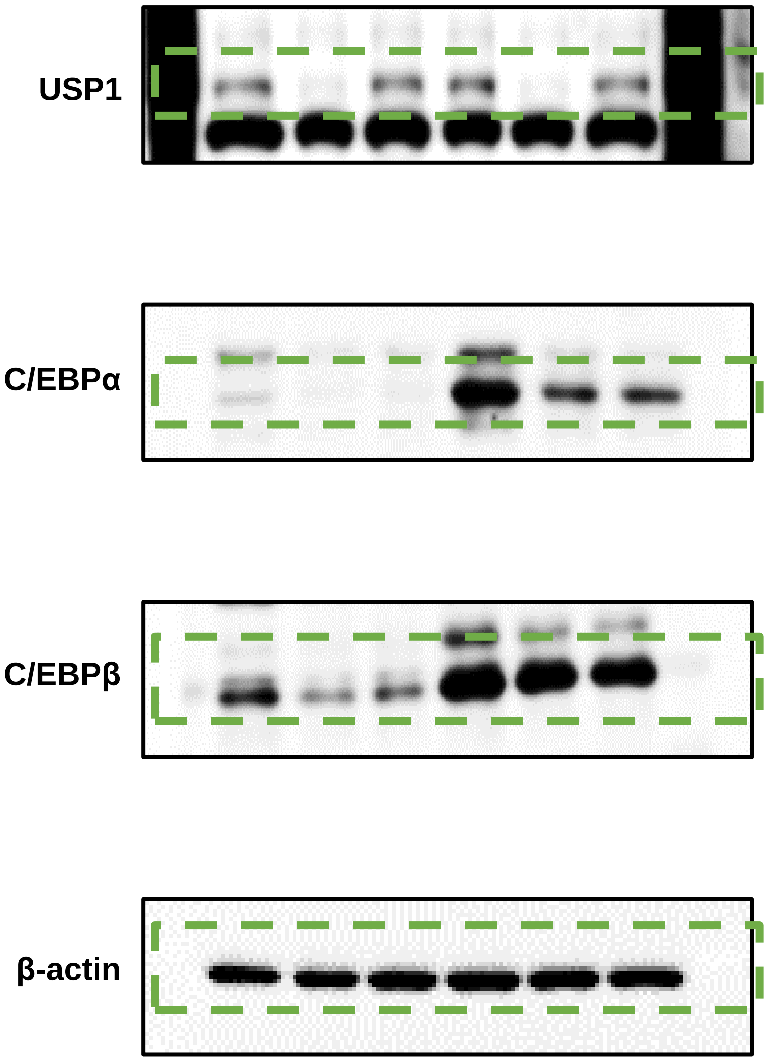

Figure 4C

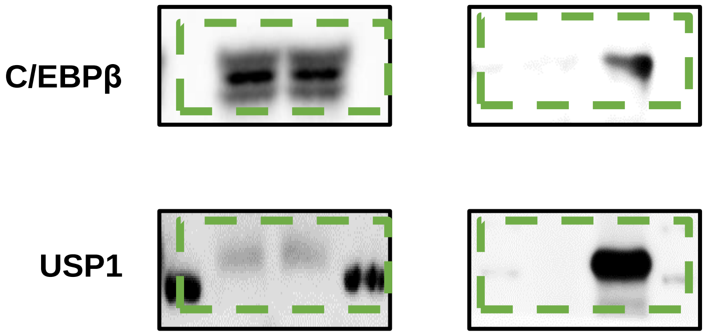

Figure 4D

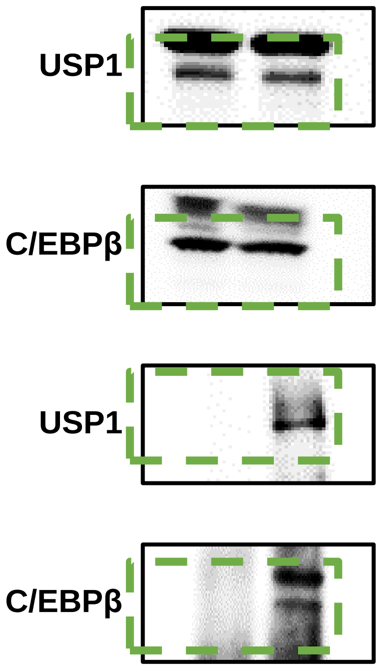

Figure 4E

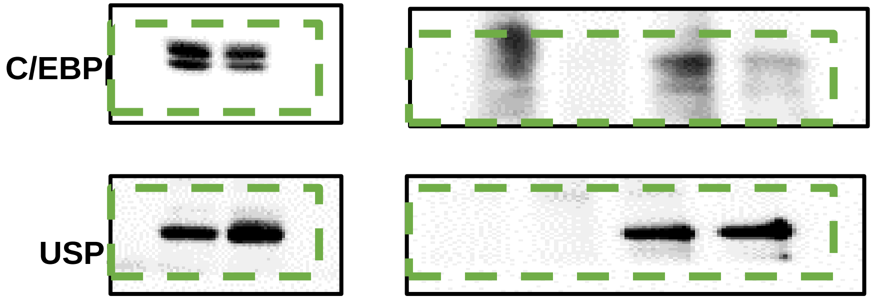

Figure 4F

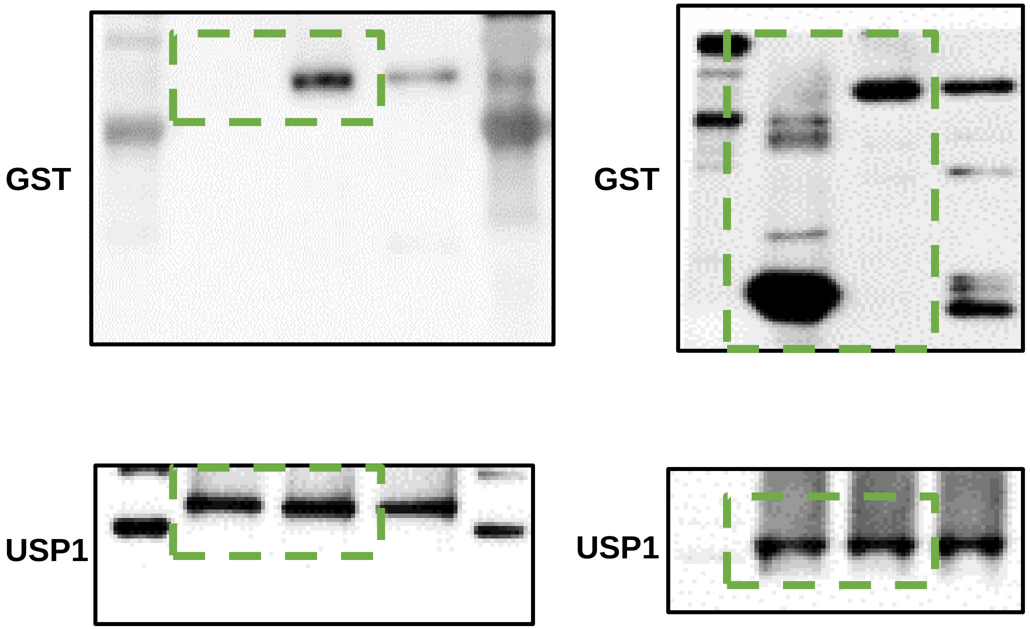

Figure 4G

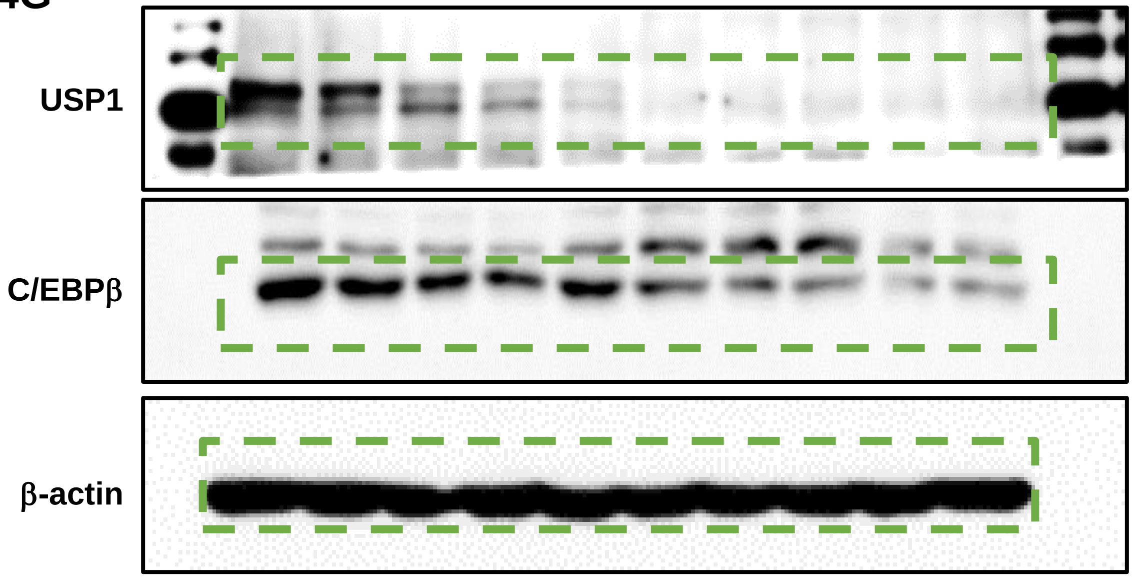

Figure 4H

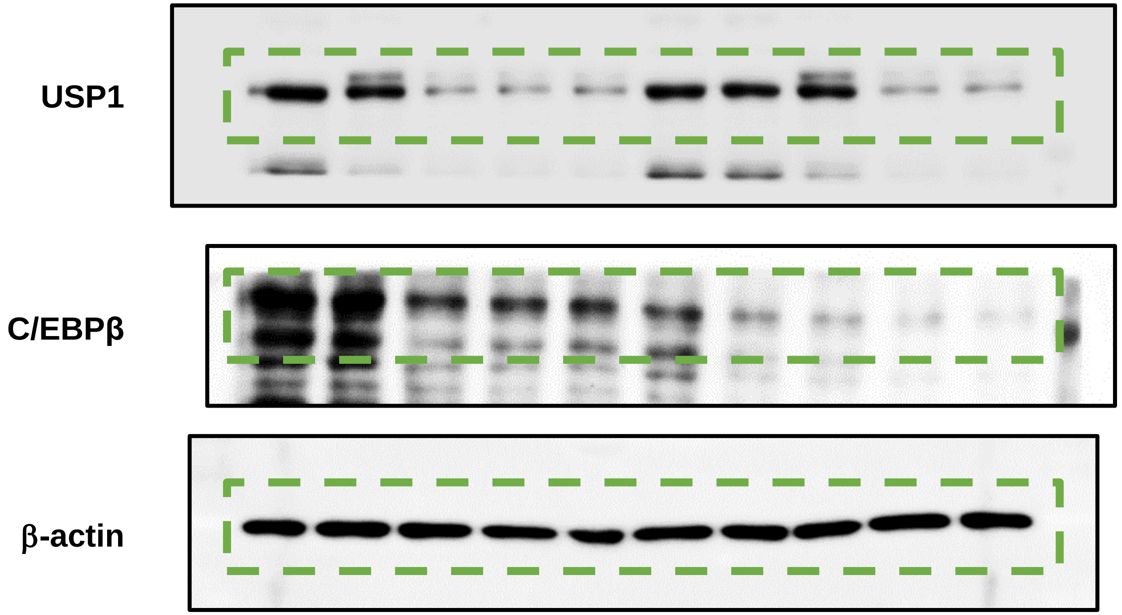

Figure 5A

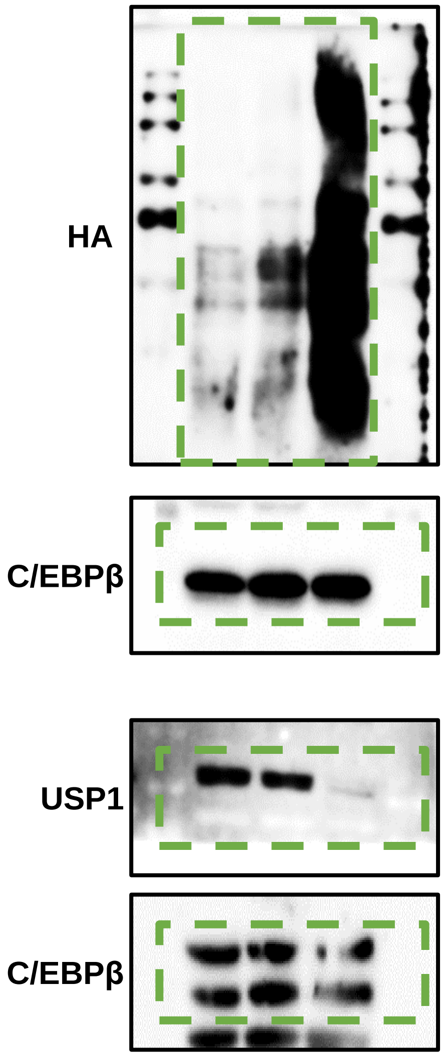

Figure 5B

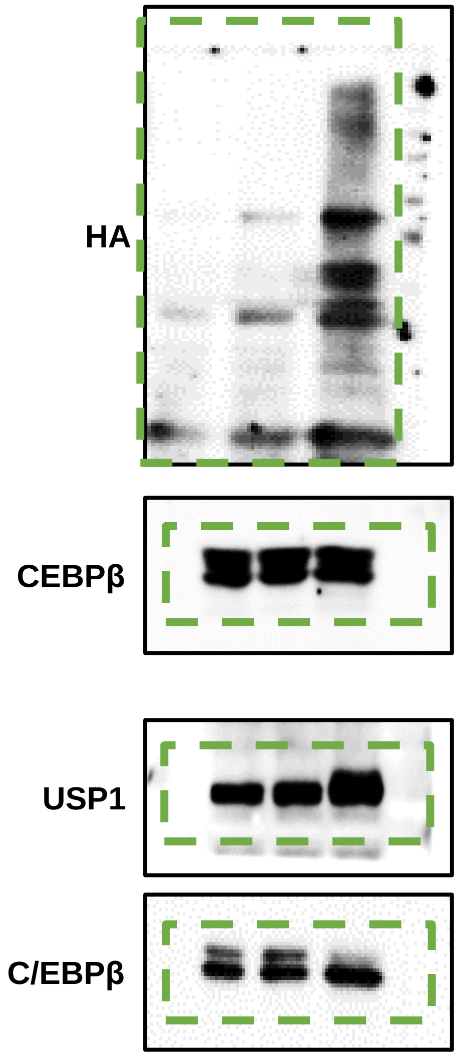

Figure 5C

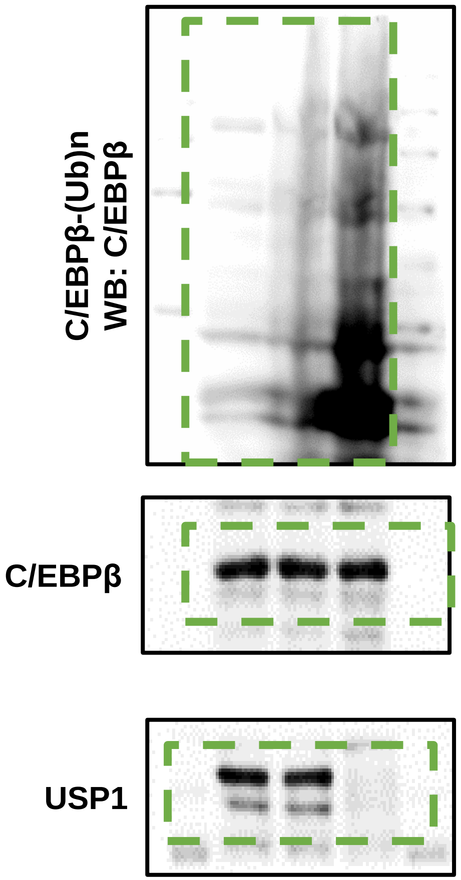

Figure 5D

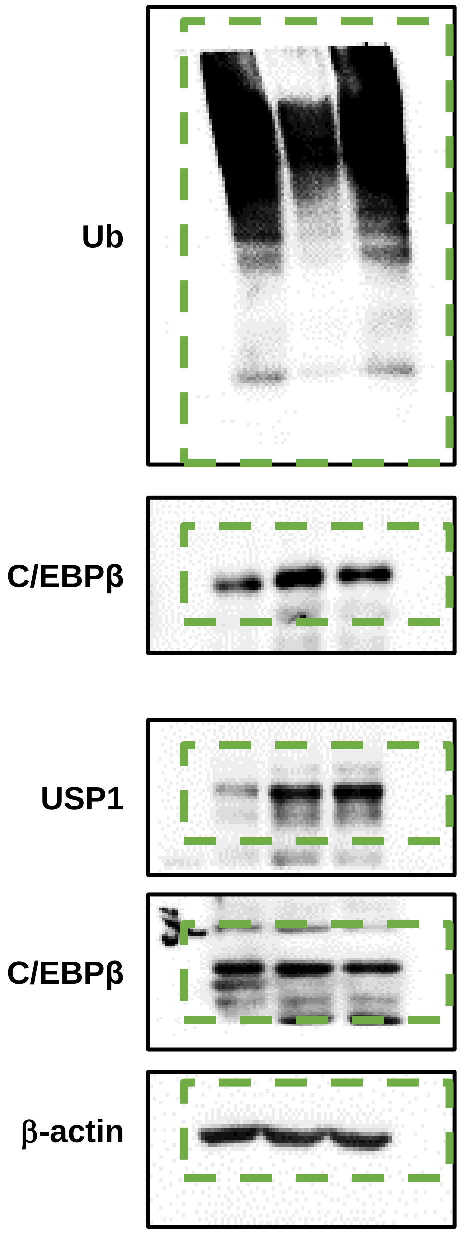

Figure 5E

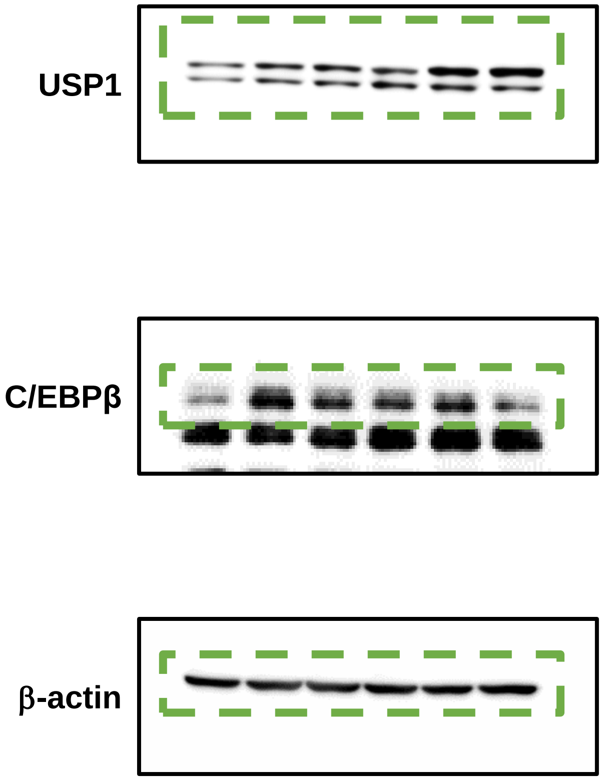

Figure 5F

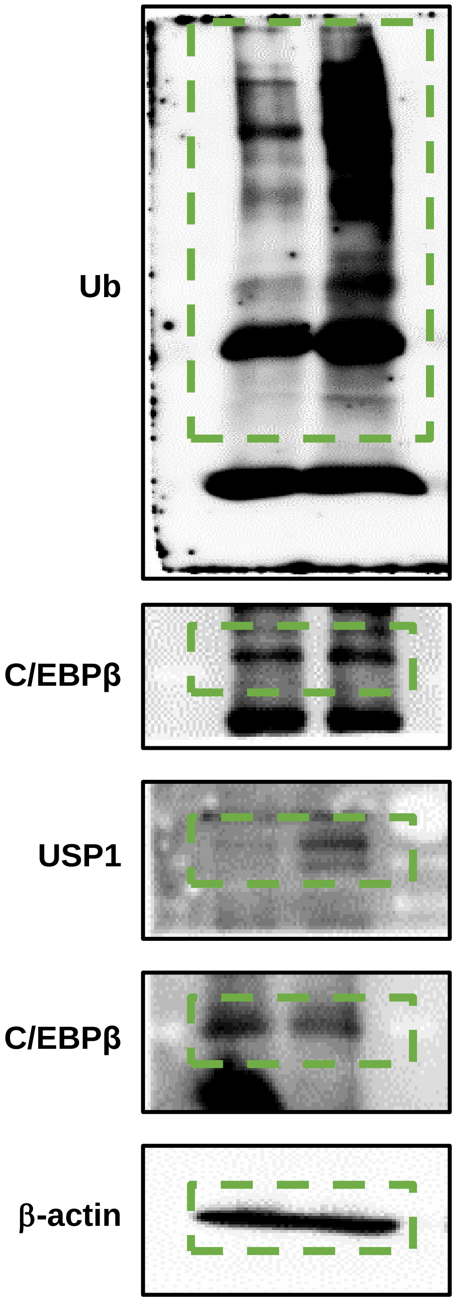

Figure 5G

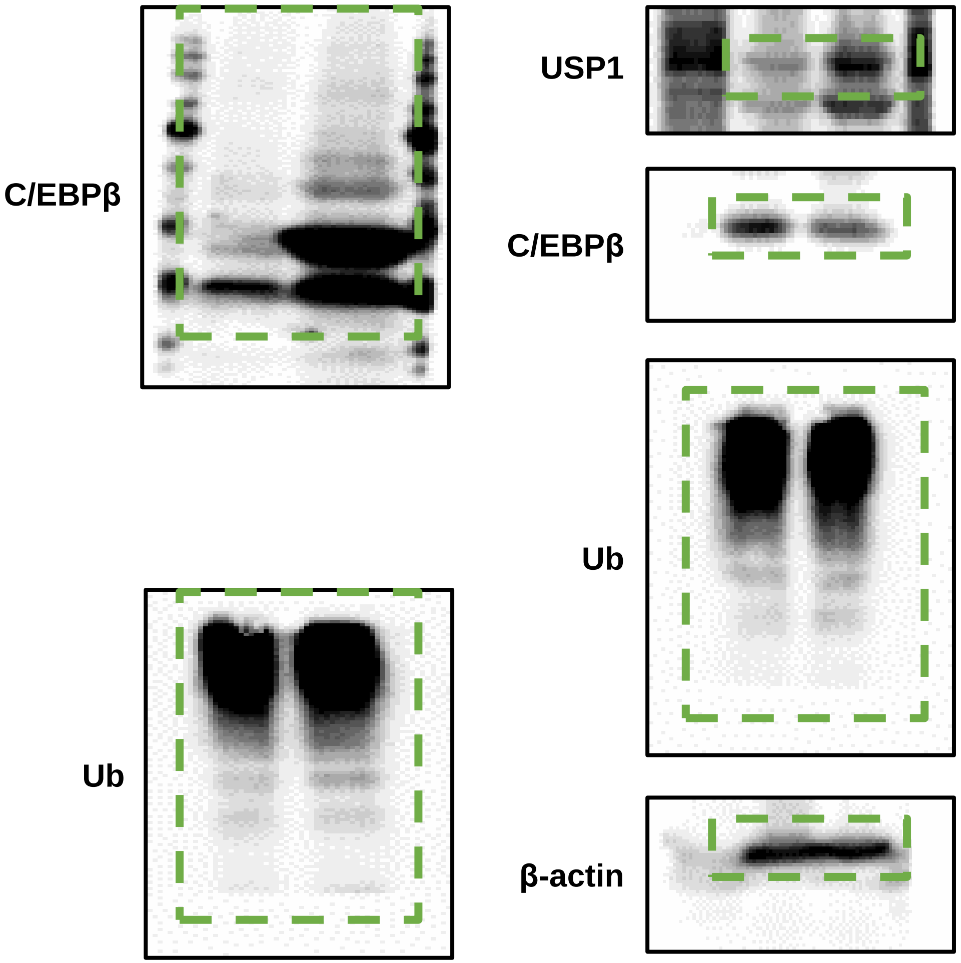

Supplementary Figure 2

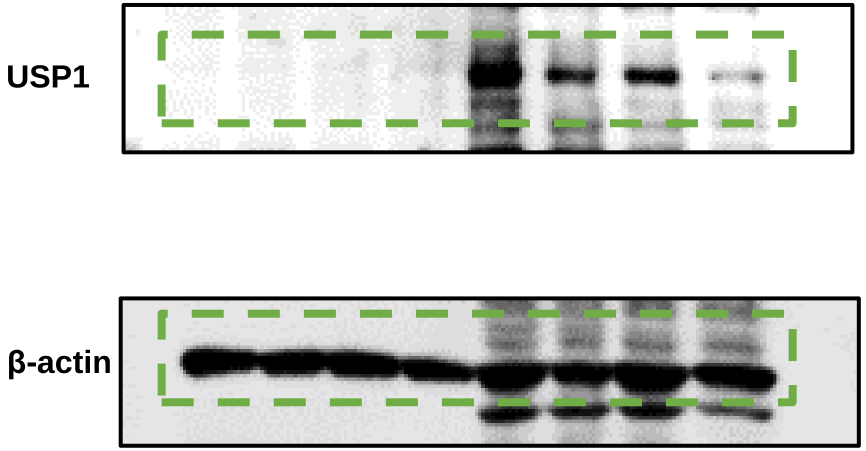

Supplementary Figure 7

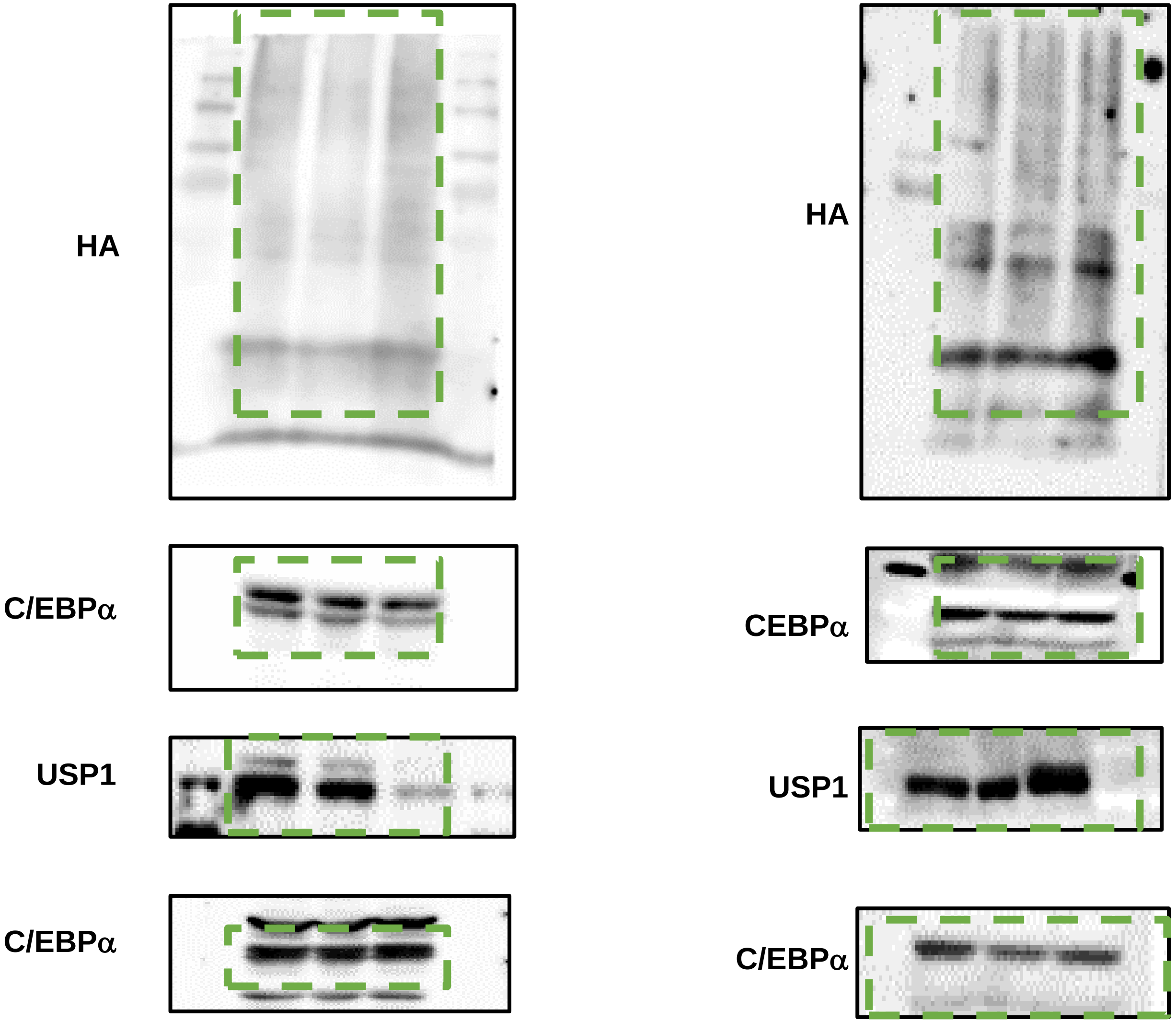

Supplementary Figure 8

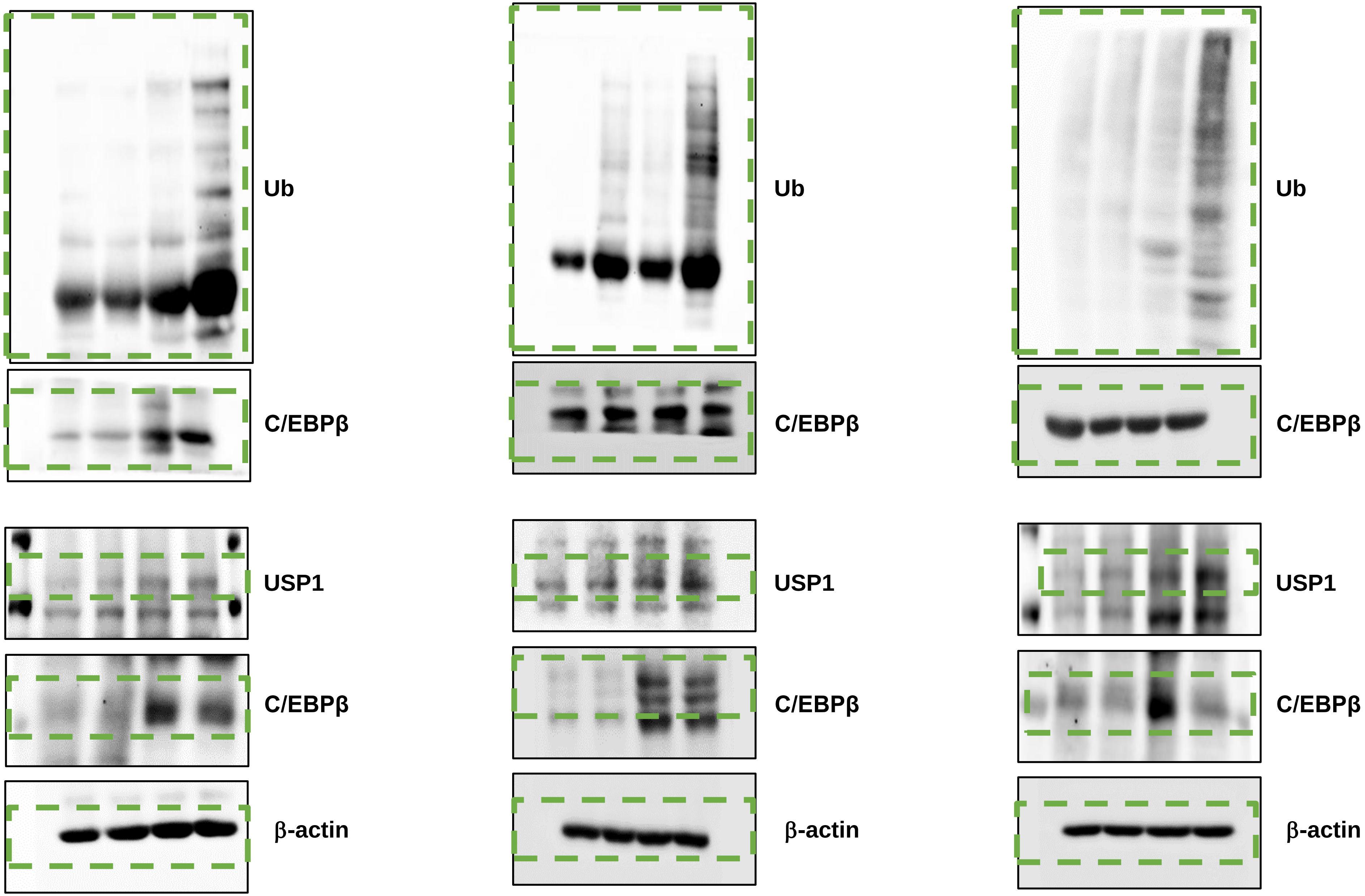

Supplement: Supplementary file 2 — Original Data File [file 41419_2023_6317_MOESM2_ESM.pdf]
